# BAMLET administration via drinking water inhibits intestinal tumor development and promotes long-term health

**DOI:** 10.1038/s41598-024-54040-w

**Published:** 2024-02-15

**Authors:** Hien Thi Tran, Murphy Lam Yim Wan, Ines Ambite, Michele Cavalera, Mario Grossi, Jaromir Háček, Parisa Esmaeili, António N. B. M. Carneiro, Arunima Chaudhuri, Shahram Ahmadi, Catharina Svanborg

**Affiliations:** 1 Division of Microbiology, Immunology and Glycobiology, Department of Laboratory Medicine, Faculty of Medicine, Lund University, Klinikgatan 28, 221 84 Lund, Sweden; 2 Department of Pathology and Molecular Medicine, Motol University Hospital, 2nd Faculty of Medicine, Charles University Praha, 150 06 Prague, Czech Republic

**Keywords:** Colon cancer, Cancer therapy

## Abstract

Though new targeted therapies for colorectal cancer, which progresses from local intestinal tumors to metastatic disease, are being developed, tumor specificity remains an important problem, and side effects a major concern. Here, we show that the protein-fatty acid complex BAMLET (bovine alpha-lactalbumin made lethal to tumor cells) can act as a peroral treatment for colorectal cancer. *Apc*^*Min/*+^ mice, which carry mutations relevant to hereditary and sporadic human colorectal cancer, that received BAMLET in the drinking water showed long-term protection against tumor development and decreased expression of tumor growth-, migration-, metastasis- and angiogenesis-related genes. BAMLET treatment via drinking water inhibited the Wnt/β-catenin and PD-1 signaling pathways and prolonged survival without evidence of toxicity. Systemic disease in the lungs, livers, spleens, and kidneys, which accompanied tumor progression, was inhibited by BAMLET treatment. The metabolic response to BAMLET included carbohydrate and lipid metabolism, which were inhibited in tumor prone *Apc*^*Min/*+^ mice and weakly regulated in C57BL/6 mice, suggesting potential health benefits of peroral BAMLET administration in addition to the potent antitumor effects. Together, these findings suggest that BAMLET administration in the drinking water maintains antitumor pressure by removing emergent cancer cells and reprogramming gene expression in intestinal and extra-intestinal tissues.

## Introduction

Targeting early locally growing tumors is essential to prevent tumor formation and reduce the risk for tumor progression and metastatic disease. While surgery is highly efficient and may remove the tumor, chemotherapy is often needed, depending on the tumor classification and predicted risk and whether the tumor has spread to the lymph nodes prior to surgery^1^. Chemotherapeutic drugs also target healthy tissues, so the treatment benefits of chemotherapy have to be weighed against the risks of toxicity and associated morbidity.

Colorectal cancer is a leading cause of death, and > 180,000 cases of colorectal cancer are diagnosed annually in the US^2^. Genetic predisposition is a risk factor, and mutations of the adenomatous polyposis coli (*APC*) gene can cause both classic and attenuated familial adenomatous polyposis^3^. The *APC* gene and Wnt/β-catenin signaling network regulate intestinal cell growth and physiology, and loss-of-function mutations in *APC* may result in cell overgrowth and polyp formation^4^. *Apc*^*Min/*+^ mice carry an *Apc* allele with a nonsense point mutation and develop tumors early in life and progressive disease due to a significant tumor burden^5^. Multiple intestinal neoplasia (Min) are detected already at 10 weeks of age and tumors usually develop in all mice, resulting in severe disease around 17 weeks of age, depending on dietary factors^6,7^.

BAMLET (bovine alpha-lactalbumin made lethal to tumor cells) is a complex formed by partially unfolded bovine alpha-lactalbumin and oleic acid and belongs to a new class of tumoricidal molecules with documented cancer specificity^8–14^. Complexes derived from the milk protein alpha-lactalbumin have strong tumoricidal properties and limited toxicity in animal models and clinical trials^15,16^. HAMLET (human alpha-lactalbumin made lethal to tumor cells) effectively kills a wide range of tumor cells and has shown therapeutic efficacy in several cancer models and clinical studies^17–24^. A second HAMLET family member, alpha1-oleate, formed by the N-terminal alpha-helical peptide of alpha-lactalbumin, has shown therapeutic efficacy in patients with bladder cancer, in controlled clinical trials^25^.

In this study, we aimed to investigate whether BAMLET can be used as a peroral therapeutic using the *Apc*^*Min/*+^ mouse model of intestinal cancer. Therapeutic end points were intestinal tumor progression and disease severity. Molecular disease determinants were identified by gene expression analysis and staining for specific tissue markers. In addition, unexpected effects on extra-intestinal organs were observed and evaluated.

## Results

### Peroral BAMLET treatment reduces intestinal tumor progression

**Figure 1:**
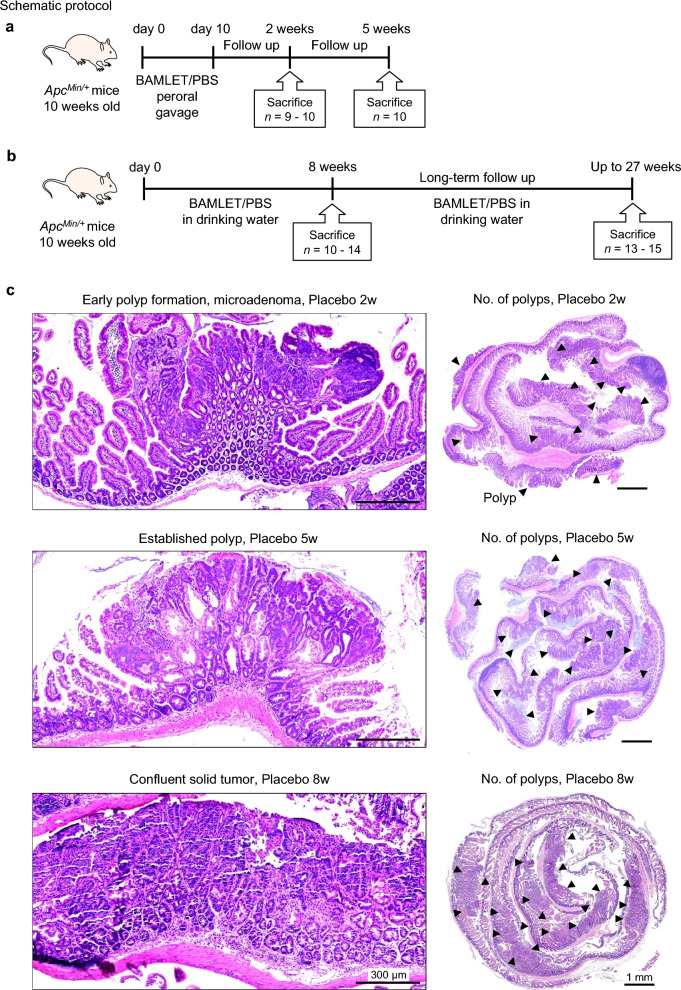

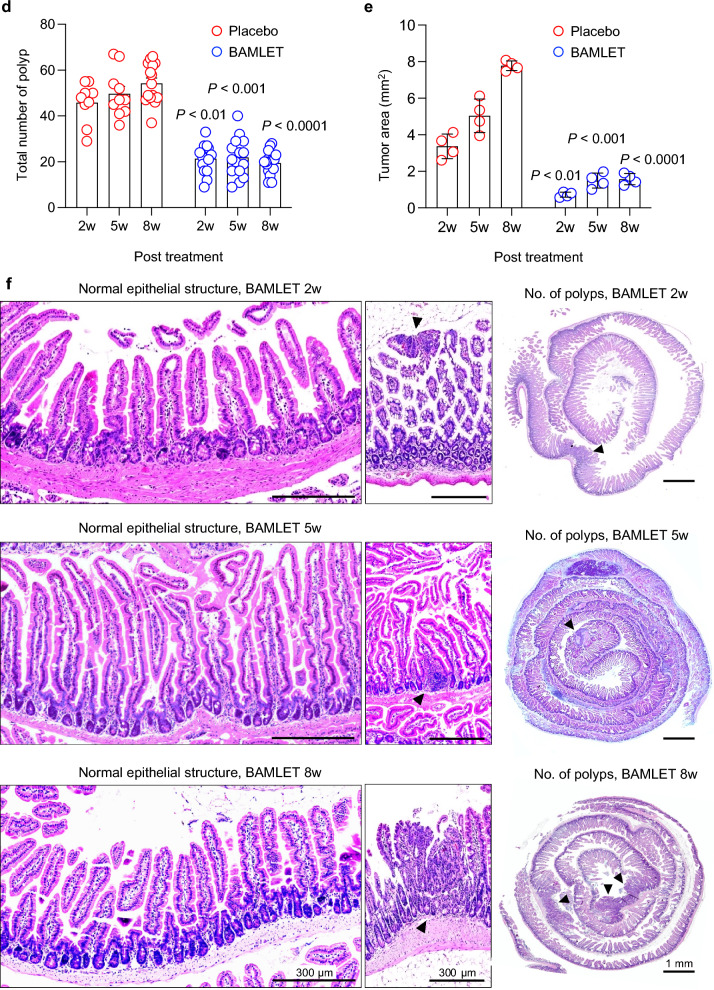
Protective effect of peroral BAMLET treatment against intestinal cancer in *Apc*^*Min/*+^ mice. (**a**) Schematic representation of the peroral treatment models. Ten-week-old female *Apc*^*Min/*+^ mice received 20 mg of BAMLET in 2 × 200 μl PBS (BAMLET) or 2 × 200 μl of PBS alone (placebo) by daily gavage for ten days. The mice were sacrificed two weeks (2w, two experiments; PBS/placebo: *n* = 9, BAMLET: *n* = 10) or five weeks (5w, two experiments; PBS/placebo: *n* = 10, BAMLET: *n* = 10) after the end of treatment (2w or 5w post-treatment). (**b**) Ten-week-old female mice received 20 mg of BAMLET in 5 ml PBS or PBS alone, daily in the drinking water until sacrifice after eight weeks (8w, two experiments; PBS/placebo: *n* = 10, BAMLET: *n* = 14) or long-term follow-up (two experiments; PBS/placebo: *n* = 13, BAMLET: *n* = 15). (**c**) H&E-stained intestinal sections showing the progression of intestinal cancer in the placebo group from microadenomas (2w), to polyps (5w), and confluent solid tumors (8w). Representative sections, *n* = 4–5 mice per group. Polyps are indicted by the arrowheads. (**d**) The total number of solid tumors per intestine was significantly reduced in the BAMLET-treated *Apc*^*Min/*+^ mice. (**e**) The tumor areas were significantly reduced in the BAMLET-treated *Apc*^*Min/*+^ mice compared to the placebo group, quantified in Swiss roll sections (*n* = 4 mice per group). (**f**) H&E-stained intestinal sections obtained from the BAMLET-treated mice after two, five and eight weeks. Data are presented as the means ± S.E.M.s from two experiments and were analyzed by two-way ANOVA with Šídák's multiple comparisons test, the BAMLET-treated mice compared to the placebo group (**d**,**e**). Scale bars = 300 μm (close-up), 1 mm (whole tissue mount) (**c**,**f**).

This study investigated how the BAMLET complex affects tumor development and tumor progression in *Apc*^*Min/*+^ mice, which carry mutations relevant to hereditary and sporadic human colorectal cancer and develop intestinal polyps that progress to form large tumors^6,26^. Two treatment protocols were used to administer BAMLET into the intestinal tract of *Apc*^*Min/*+^ mice (10 weeks old, Fig. 1a,b). According to protocol 1, mice were subjected to peroral BAMLET gavage twice daily for ten days (Fig. 1a) and sacrificed two- or five-weeks post-treatment. According to protocol 2, mice were continuously administered BAMLET in drinking water and were sacrificed after eight weeks or followed long-term until their health deteriorated (Fig. 1b). Control mice received drinking water supplemented with phosphate buffered saline salts (PBS, placebo group).

In this study, all *Apc*^*Min/*+^ mice in the placebo group developed intestinal tumors. Tumor progression was quantified as the increase in tumor number and size by dissection microscopy and was confirmed by high-resolution imaging of hematoxylin–eosin (H&E)-stained tissue sections; specifically, an increase in tumor size from microadenomas (two weeks) and fully formed macroadenomas (five weeks) to solid tumors (eight weeks) occupying most of the intestinal wall was observed (Fig. 1c). Polyps were counted in all groups by visual inspection of the whole length of the intestine and emerging tumors were identified by histopathology of intestinal tissue sections (Fig. 1d,e). The gradual increase in polyp number and tumor area was further visualized by “Swiss-roll” preparations of intestinal segments and methylene blue staining (Fig. 1, Supplementary Fig. S1).

Peroral BAMLET treatment reduced the number of small tumors, fully formed polyps and confluent tumors along the intestinal wall, compared to the placebo group (Fig. 1f and Supplementary Fig. S1). A normal villus structure predominated after short-term BAMLET gavage and the tumor number and tumor area were significantly lower after ten days of oral BAMLET gavage and a follow up of two or five weeks, suggesting a rapid and lasting treatment effect (Fig. 1f and Supplementary Fig. S1). BAMLET administration in the drinking water showed a strong protective effect after eight weeks with a marked reduction in polyp number and polyp size compared to the placebo group (Fig. 1d–f and Supplementary Fig. S1). The results suggest a potent anti-tumor effect of BAMLET, preventing tumor establishment and progression.

### Inhibition of cancer-related gene expression by BAMLET treatment

**Figure 2:**
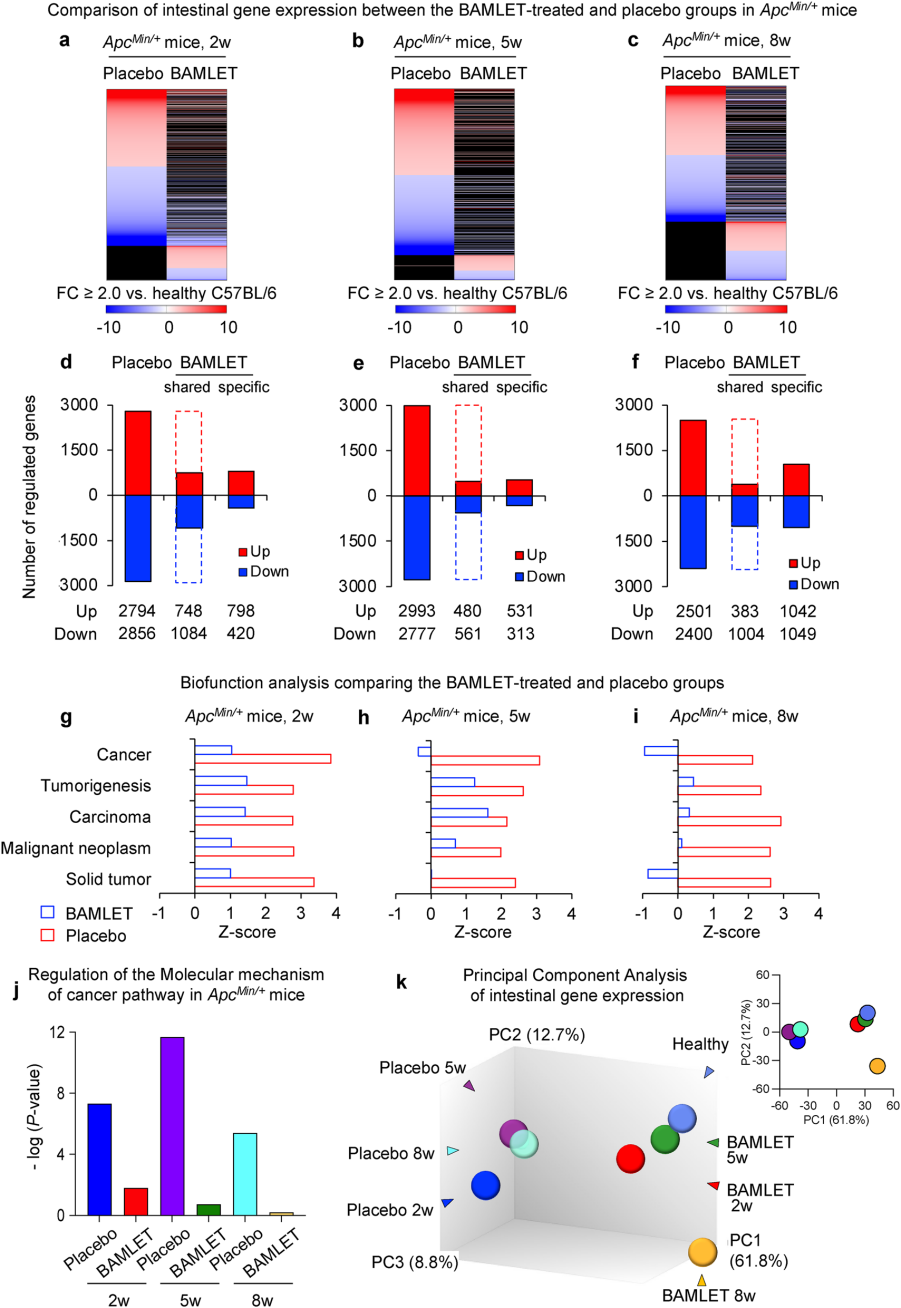
Gene expression in *Apc*^*Min/*+^ developing progressive intestinal disease, compared to healthy mice. (**a**–**c**) Heatmaps comparing gene expression profiles between the placebo-treated *Apc*^*Min/*+^ mice and mice administered BAMLET by gavage (**a**,**b**) or in drinking water (**c**). Mice were sacrificed two weeks (2w) (**a**), five weeks (5w) (**b**) or eight weeks (8w) (**c**) post treatment (red: upregulated genes, blue: downregulated genes, black: unregulated genes, cutoff FC ≥ 2.0 compared to gene expression in healthy intestinal tissue from untreated C57BL/6 mice, *n* = 1 RNA sample per group). (**d**–**f**) Histograms showing the number of regulated genes in the placebo-treated *Apc*^*Min/*+^ mice and the mice that received BAMLET at 2w (**d**), 5w (**e**) or 8w (**f**). (**g**–**i**) Biofunction analysis of cancer-related biofunctions in the BAMLET-treated group 2w (**g**), 5w (**h**) or 8w (**i**) post treatment compared to the placebo-treated group. (**j**) Regulation of cancer pathway genes in the placebo-treated *Apc*^*Min/*+^ mice and the mice that received BAMLET, 2w, 5w and 8w. **k**, Principal component analysis of mRNA profiles of intestinal tissue segments. The mice treated with BAMLET formed a cluster near the cluster corresponding to the healthy C57BL/6 mice and distant from the cluster corresponding to the placebo-treated *Apc*^*Min/*+^ mice.

To characterize the disease response and how it is affected by BAMLET treatment, total intestinal RNA was subjected to whole-genome transcriptomic profiling. The gene expression profiles of samples from the placebo-treated *Apc*^*Min/*+^ mice with growing tumors and the BAMLET-treated *Apc*^*Min/*+^ mice were first compared to healthy C57BL/6 mice, in which intestinal tumors do not grow (Fig. 2). The expression of a large number of genes was altered in the placebo-treated *Apc*^*Min/*+^ mice and the biofunctions of the top differentially expressed genes were cancer-related, compared to the healthy C57BL/6 mice, in which intestinal tumors do not grow (Fig. 2a–i). In contrast, gene expression was significantly less affected in the BAMLET-treated *Apc*^*Min/*+^ mice, including the cancer-related biofunctions, which predominated in the placebo group (Fig. 2a–i). This inhibitory effect involved genes in the cancer gene network (Fig. 2j) and colon cancer-related genes (Supplementary Fig. S2). Principal component analysis (PCA) showed that the genes expressed in BAMLET-treated *Apc*^*Min/*+^ mice formed a cluster near the healthy mice and distant from the cluster corresponding to the placebo-treated *Apc*^*Min/*+^ mice (Fig. 2k), consistent with the observation that the BAMLET-treated mice did not develop tumors to the same extent and were protected from the rapid and severe disease observed in the placebo group and remained more healthy.

### Mechanism of tumor inhibition in BAMLET-treated ApcMin/+ mice

**Figure 3:**
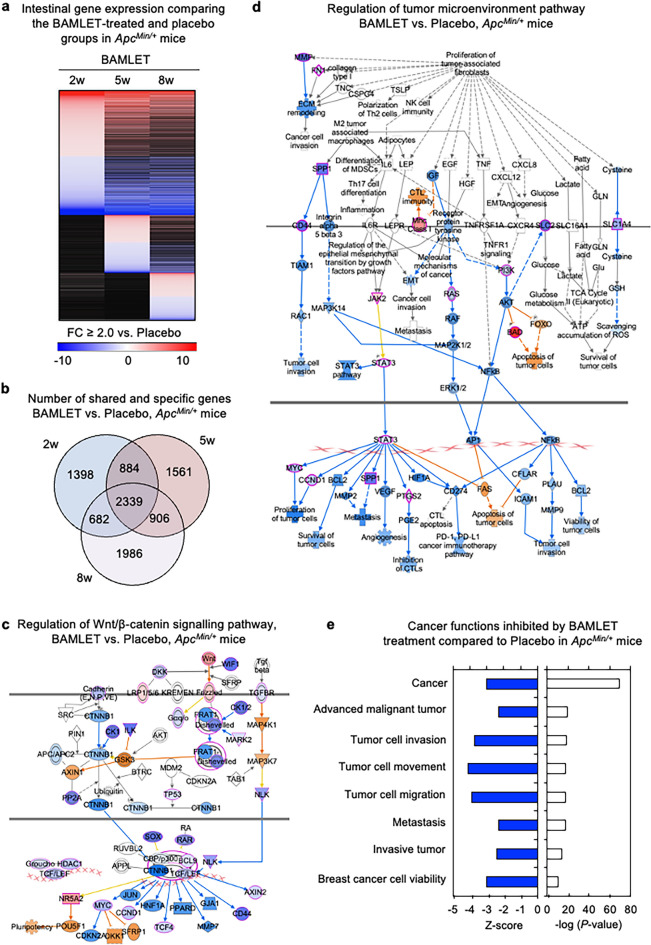
Mechanism of BAMLET inhibition, defined by a direct comparison of gene expression in intestinal tissues from placebo-treated or BAMLET-treated *Apc*^*Min/*+^ mice. (**a)** Heatmap directly comparing intestinal gene expression profiles in *Apc*^*Min/*+^ mice treated with BAMLET (10 mg/200 μl) or placebo (200 μl of PBS) twice daily by oral gavage (red: upregulated genes, blue: downregulated genes, black: nonregulated genes, cutoff FC ≥ 2.0 BAMLET compared to the placebo group for each time point, *n* = 1 RNA sample per group). **(b**) Venn diagram identifying genes regulated at all time points, in the three BAMLET treatment groups compared to the placebo-treated group (*n* = 2,339 genes). (**c**,**d)** Network analysis of shared genes (*n* = 2,339) revealed major treatment effects. (**c)** Wnt/β-catenin signaling was inhibited in all the BAMLET-treated *Apc*^*Min/*+^ mice. (**d**) Genes in the tumor microenvironment network were broadly inhibited, potentially reducing proliferation, angiogenesis, metastasis, and the PD-1 pathway. Blue and orange represent inhibition and activation, whereas red and blue represent upregulation and downregulation, respectively. (**e**) Cancer-related biofunctions such as tumor growth, cell movement, invasion and metastasis were inhibited. Enrichment *P*-values and activation Z-scores for individual pathways are indicated.

To further analyze the mechanism by which BAMLET protects against intestinal cancer, the gene expression profiles were directly compared between the BAMLET-treated *Apc*^*Min/*+^ mice and the placebo-treated *Apc*^*Min/*+^ mice at each time point (Fig. 3). Strong treatment effects were documented in mice receiving BAMLET by oral gavage or in the drinking water (Fig. 3a,b and Supplementary Fig. S3). Regulated genes shared by the three groups included genes in the Wnt/β-catenin signaling pathway, which were inhibited (Fig. 3c) as well as genes that define the tumor microenvironment (Fig. 3d). Ingenuity pathway analysis identified cancer-related biofunctions as strongly inhibited, predicted to affect angiogenesis, tumor cell mobility, tumor growth, metastasis and specifically colon cancer metastasis (Fig. 3e).

To confirm these effects at the protein level, intestinal tissue sections were stained for the tumor markers vascular endothelial growth factor (VEGF), Ki67, cyclin D1 and β-catenin (Supplementary Fig. S4). Sections were obtained from the placebo-treated mice or mice receiving BAMLET by oral gavage after five weeks follow-up or in the drinking water for eight weeks (Supplementary Fig. S4). VEGF, Ki67, cyclin D1 or β-catenin staining was reduced at both time points in the BAMLET-treated *Apc*^*Min/*+^ mice (Supplementary Fig. S4), consistent with the observation that these mice did not show tumor progression.

### Long-term protection against tumor development and increased survival in BAMLET-treated mice

**Figure 4:**
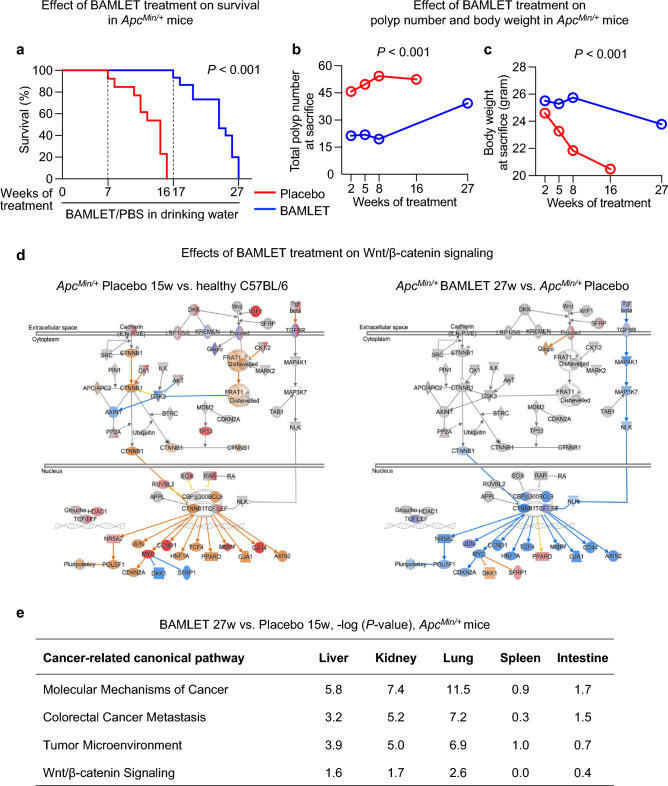
Long-term protection by BAMLET administration via drinking water. Schematic of the protocol is shown in Fig. 1b. (**a**) *Apc*^*Min/*+^ mice received drinking water supplemented with BAMLET (20 mg in 5 ml PBS daily) or PBS (placebo, 5 ml PBS daily) from ten weeks of age (10w). Mice in the placebo group developed severe disease and all were sacrificed after 16 weeks (16w). BAMLET supplementation increased the survival time by about 10w and all of the BAMLET treated mice were sacrificed after 27 weeks (27w). (**b**) Time dependent effect on the total polyp number and (**c**), body weight in the BAMLET-treated mice compared to the placebo-treated *Apc*^*Min/*+^ mice. BAMLET-treated mice, two experiments,* n* = 15; placebo-treated mice, two experiments,* n* = 13. (**d**) Gene expression analysis identified the Wnt/β-catenin signaling pathway as activated in the placebo-treated *Apc*^*Min/*+^ mice (15 weeks of PBS in drinking water), but these genes were inhibited or not regulated in the BAMLET-treated *Apc*^*Min/*+^ mice (27 weeks of BAMLET in drinking water). Blue and orange represent inhibition and activation, whereas red and blue represent upregulation and downregulation, respectively. *n* = 2 RNA samples per group. (**e**) Cancer-related canonical pathways regulated in the BAMLET-treated mice compared to the placebo-treated mice at long-term follow-up. Genes in the cancer pathway were strongly regulated by BAMLET treatment, as well as genes related to colorectal cancer metastasis, the tumor microenvironment and the Wnt/β-catenin signaling pathway.

Supplementation of the drinking water with BAMLET was shown to have a lasting protective effect against tumor formation and tumor progression. To determine whether this effect would translate to a beneficial effect on survival, mice were followed until symptoms became evident (weight loss > 20%, hunched back, prostration, low response to stimulation and lack of grooming). As shown by Kaplan‒Meier analysis, a significant increase in survival was detected in the BAMLET-treated *Apc*^*Min/*+^ mice compared to the placebo group (Fig. 4a). While mice in the placebo-treated group survived up to 16 weeks, the BAMLET-treated group survived for up to 27 weeks, which is eleven weeks longer than the placebo-treated group. Long-term BAMLET treatment for up to 27 weeks reduced the total polyp number and polyp size and prevented the loss of body weight that accompanies the progression of intestinal disease in the placebo-treated *Apc*^*Min/*+^ mice (Fig. 4b,c).

### Long-term effects on intestinal gene expression

The intestinal gene expression profiles of the placebo-treated *Apc*^*Min/*+^ mice and BAMLET-treated *Apc*^*Min/*+^ mice differed substantially from one another. This included a difference in the expression of genes in the Wnt/β-catenin signaling pathway, which was upregulated in the placebo-treated *Apc*^*Min/*+^ mice compared to the healthy C57BL/6 mice and downregulated in the BAMLET-treated *Apc*^*Min/*+^ mice compared to the placebo-treated *Apc*^*Min/*+^ mice (Fig. 4d,e, Supplementary Fig. S8). Further support for this difference in gene expression was obtained by intestinal β-catenin staining, which was strongly reduced in the BAMLET-treated *Apc*^*Min/*+^ mice compared to the placebo group (Supplementary Fig. S7), confirming the inhibition of the Wnt/β-catenin pathway by BAMLET at the protein level.

**Figure 5:**
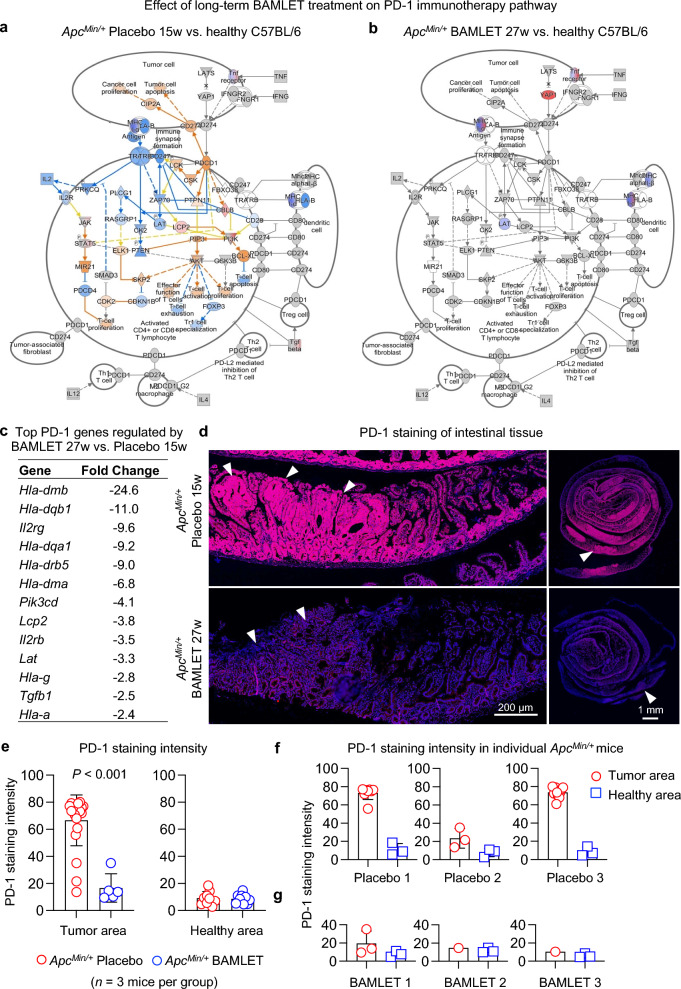
Inhibition of PD-1 signaling by BAMLET administration in the drinking water. (**a**) Gene expression analysis of intestinal RNA identified the PD-1 pathway as strongly enhanced in the placebo-treated *Apc*^*Min/*+^ mice compared to the healthy C57BL/6 mice at long-term follow-up (cutoff FC ≥ 2, *P* < 0.05, *n* = 2 RNA samples per group). (**b**) Genes in the PD-1 pathway were not regulated in the BAMLET-treated *Apc*^*Min/*+^ mice compared to the healthy C57BL/6 mice. Blue and orange represent inhibition and activation, whereas red and blue represent upregulation and downregulation, respectively. (**c)** Genes in the PD-1 pathway were inhibited in the BAMLET-treated mice compared to the placebo-treated *Apc*^*Min/*+^ mice. (**d**) PD-1 staining of intestinal sections from the placebo-treated or BAMLET-treated *Apc*^*Min/*+^ mice was performed with anti-PD-1 antibodies (magenta = PD-1, blue = DAPI). Representative sections, *n* = 3 mice per group. Arrows in the Swiss roll preparation indicate the position of the tumor areas magnified. (**e)** PD-1 staining in the Swiss roll preparation was quantified, comparing tumor areas to healthy areas in the placebo-treated and the BAMLET-treated *Apc*^*Min/*+^ mice. (**f**,**g**) Quantification of PD-1 staining in intestinal sections of individual mice comparing tumor areas to healthy areas. Data are presented as the means ± S.E.M.s, *n* = 3 mice per group. Scale bars = 200 μm (close-up), 1 mm (whole tissue) (**d**).

The analysis further showed that the programmed death receptor 1 (PD-1) signaling pathway was activated in the placebo-treated *Apc*^*Min/*+^ mice compared to the healthy C57BL/6 mice (Fig. 5a). Affected genes included the T-cell-related genes *Rasgrp1*, *Cblb*, and *Lcp2*. In contrast, the BAMLET-treated *Apc*^*Min/*+^ mice showed no evidence of PD-1 pathway activation compared to the healthy C57BL/6 mice (Fig. 5b). In a direct comparison of the BAMLET-treated *Apc*^*Min/*+^ mice and the placebo group, genes in the PD-1 pathway were identified as the most strongly downregulated by BAMLET, specifically HLA class II histocompatibility antigens (*Hla-dmb, Hla-dqb1, Hla-dqa1, Hla-drb5* and *Hla-dma*), IL2 receptors (*IL2rg, Il2rb*) and the growth factor (*Tgfb1*) was downregulated to a greater extent in the BAMLET-treated *Apc*^*Min/*+^ mice compared to the placebo-treated *Apc*^*Min/*+^ mice (Fig. 5c). Furthermore, PD-1 tissue staining was significantly lower in sections from the BAMLET-treated *Apc*^*Min/*+^ mice than in the placebo-treated *Apc*^*Min/*+^ mice and was more pronounced in tumor areas than in adjacent healthy tissues (Fig. 5d–g and Supplementary Fig. S5).

### Evidence of protection against extra-intestinal disease in BAMLET-treated mice

At sacrifice, the placebo group further showed evidence of severe extra-intestinal disease, defined by macroscopic changes in major organs including the lungs, livers, spleens and kidneys. The intestines and spleen were enlarged, the livers and kidneys were discolored, and the lungs were pale and appeared more solid in the placebo-treated *Apc*^*Min/*+^ mice compared to the healthy C57BL/6 mice (Supplementary Fig. S6). The spleen weight increased, and the liver weight increased relative to the whole-body weight in the placebo group (Supplementary Fig. S6). These changes were much less pronounced in the *Apc*^*Min/*+^ mice received BAMLET in the drinking water, except for moderate enlargement of the spleens of individual mice (Supplementary Fig. S6). The BAMLET-treated group continued to gain weight, unlike the placebo group (Fig. 4c).

The tissues were further examined by histopathology. Lung tissues from the placebo-treated mice showed thickened alveolar septa and reduced alveolar spaces, suggesting hypercellularity or focal collapse of the lung parenchyma (Supplementary Fig. S6). The liver tissue showed evidence of centrilobular micro- and macrovacuolar steatosis or “fatty liver”, and binucleated hepatocytes were observed. The spleens of *Apc*^*Min/*+^ mice in the placebo group showed the loss of lymphoid foci and a more chaotic arrangement of lymphoid cells than those of the healthy C57BL/6 mice, which do not carry the *Apc*^*Min/*+^ mutation or develop intestinal tumors (Supplementary Fig. S6).

**Figure 6:**
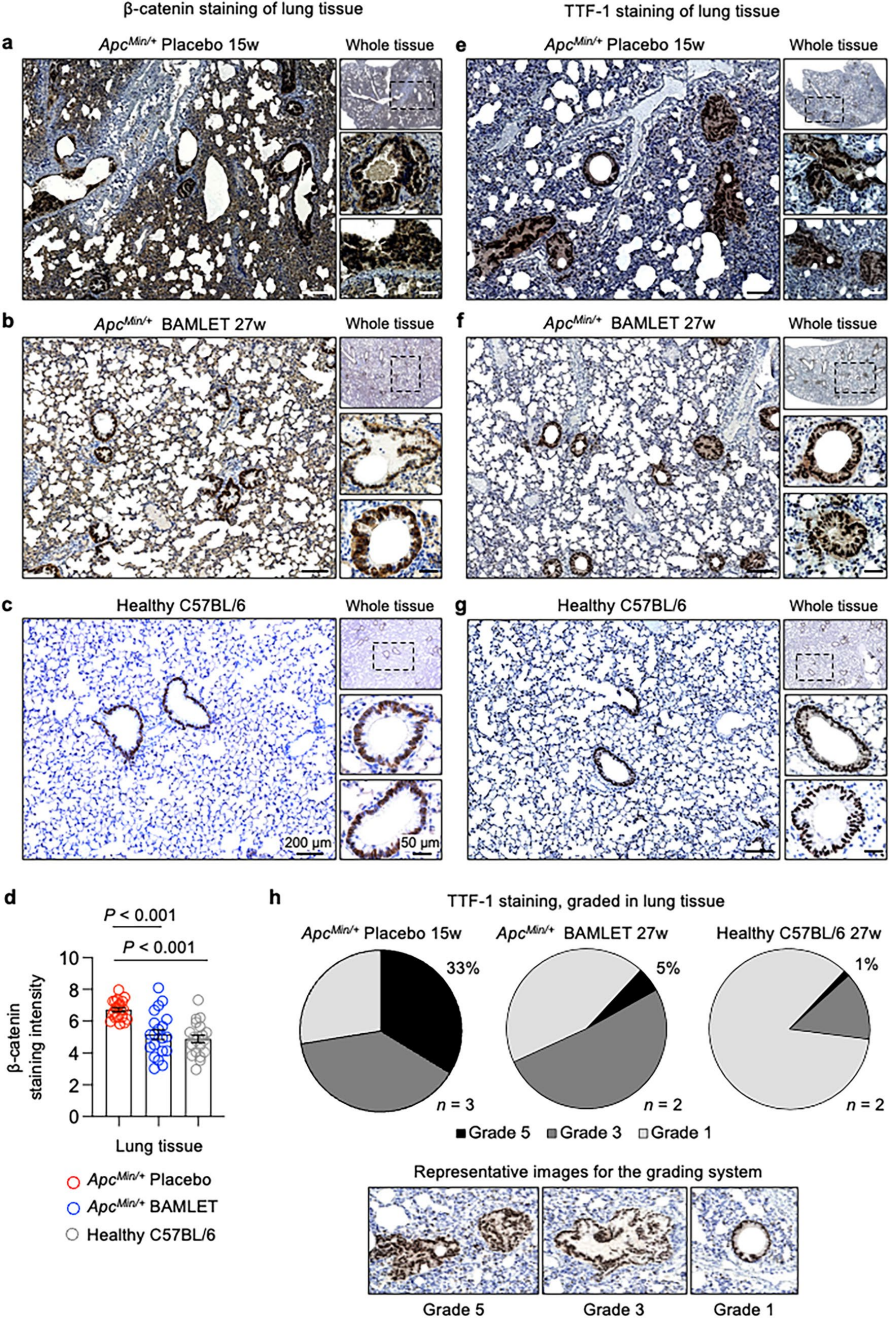
BAMLET administration via drinking water inhibits β-catenin and lung cancer related markers and promotes long-term health. (**a**–**c**) β-catenin staining of lung tissue sections from the placebo-treated, BAMLET-treated *Apc*^*Min/*+^ mice or healthy C57BL/6 mice, examined by immunohistochemistry, after staining with β-catenin-specific antibodies followed by H&E counterstaining. (**a**) Significant increase in overall β-catenin staining in the placebo-treated *Apc*^*Min/*+^ mice, including foci adjacent to the bronchiolar epithelium with enhanced staining. (**b**) Reduced β-catenin in the BAMLET-treated compared to the placebo-treated *Apc*^*Min/*+^ mice. (**c**) Healthy C57BL/6 mice showed a normal pattern of weak β-catenin staining in bronchial and bronchiolar epithelial cells. (**d**) Quantification of β-catenin staining in lung tissues for (**a**–**c**). Representative sections, *n* = 2 mice per group, 10 areas per mouse. Data are presented as the means ± S.E.M.s and analyzed by one-way ANOVA with Šídák's multiple comparisons test. (**e**–**g**) TTF-1 staining of lung tissue sections in the placebo-treated and BAMLET-treated *Apc*^*Min/*+^ mice or the healthy C57BL/6 mice, examined by immunohistochemistry staining with TTF1-specific antibodies followed by H&E counterstaining. Representative sections, *n* = 2–3 mice per group. € Significant increase in overall TTF-1 staining in the placebo-treated *Apc*^*Min/*+^ mice, including areas of proliferating cells with enhanced staining emanating from the bronchiolar epithelium. (**f**) Reduced TTF-1 staining in the BAMLET-treated *Apc*^*Min/*+^ mice. (**g**) C57BL/6 mice showed weak TTF-1 staining in bronchial and bronchiolar epithelial cells. (**h**) Grading of TTF-1-positive areas in whole lung sections (see inset for grading scale). Data are presented as the means, *n* = 2–3 mice per group. Scale bars = 200 μm (overview), 50 μm (close-up) (**a**–**c**,**e**–**g**).

These changes in the placebo-treated group were accompanied by a significant increase in β-catenin staining in the intestines, lungs, livers, and kidneys compared to that in the healthy C57BL/6 mice (Fig. 6a–d and Supplementary Fig. S7). Furthermore, in the placebo-treated *Apc*^*Min/*+^ mice, intense β-catenin staining of the multilayered lining of the bronchial tree and thickened septa between the alveoli was observed (Fig. 6a–c). Focal cell aggregates formed along the renal pelvis, and the overall staining intensity in the renal papillae was higher than that in the healthy C57BL/6 mice (Supplementary Fig. S7). The livers of placebo-treated *Apc*^*Min/*+^ mice also showed intense diffuse β-catenin staining (Supplementary Fig. S7). In contrast, the BAMLET-treated *Apc*^*Min/*+^ mice showed a general reduction in β-catenin staining in these tissues and focal cell aggregates stained for β-catenin were less prominent in the lungs and kidneys than in the placebo-treated mice (Fig. 6a,b and Supplementary Fig. S7). These differences in β-catenin staining between the placebo-treated and BAMLET-treated groups suggest that the treatment effects of BAMLET administration extend outside of the intestinal compartment even though BAMLET was administered in the drinking water.

By histopathology of lung tissue sections, proliferating cell foci were detected in the bronchial wall as multilayered cell clusters extending from the bronchial lumen in the placebo-treated *Apc*^*Min/*+^ mice, suggesting tumor formation (Fig. 6). Further staining was performed using antibodies against thyroid transcription factor (TTF-1), which is highly expressed in lung adenocarcinoma and used as a diagnostic marker for lung cancer^27^. TTF-1 was strongly expressed by the proliferating cells in the placebo group, and TTF-1 staining overlapped with β-catenin staining in those cell clusters (Fig. 6e–g). The number of areas with proliferating cells was markedly reduced in the BAMLET-treated *Apc*^*Min/*+^ mice, as was the level of TTF-1 staining in these areas (Fig. 6e–h).

These systemic treatment effects were confirmed by gene expression analysis comparing tissues from the placebo-treated *Apc*^*Min/*+^ mice to the BAMLET-treated *Apc*^*Min/*+^ mice (Supplementary Fig. S8). The expression of genes in the cancer gene network was upregulated in the lungs, liver, and kidneys of the placebo-treated *Apc*^*Min/*+^ mice compared to the BAMLET-treated *Apc*^*Min/*+^ mice, and differences in the expression of colon cancer metastasis-related genes and genes related to the tumor microenvironment were also observed in these organs (Supplementary Fig. S8).

This analysis identifies unexpected extra-intestinal effects of BAMLET that accompany the local effects on intestinal tumor development.

### BAMLET uptake by tumor tissue and colon cancer cells

To determine whether BAMLET was retained in the intestinal tumors, VivoTag 680-labeled BAMLET was administered to 18-week-old tumor-bearing *Apc*^*Min/*+^ mice by oral gavage and the mice were monitored, by imaging of the whole small intestine (Supplementary Fig. S9). The BAMLET-treated healthy C57BL/6 mice were used as controls. Significantly increased retention of BAMLET was detected in the tumor-bearing *Apc*^*Min/*+^ mice compared to the BAMLET-treated C57BL/6 mice after 24 and 48 h, suggesting that BAMLET was taken up by intestinal tumor tissue in vivo (Supplementary Fig. S9). Intestinal tissue sections from *Apc*^*Min/*+^ mice and C57BL/6 mice treated with BAMLET by oral gavage for 48 h were further subjected to immunohistochemistry using BAMLET-specific antibodies. A clear BAMLET- horseradish peroxidase (HRP) signal was detected after 48 h in *Apc*^*Min/*+^ mice but not in C57BL/6 mice (Supplementary Fig. S9). Detachment of tumor fragments that stained for BAMLET was detected in several sections of the intestine.

**Figure 7:**
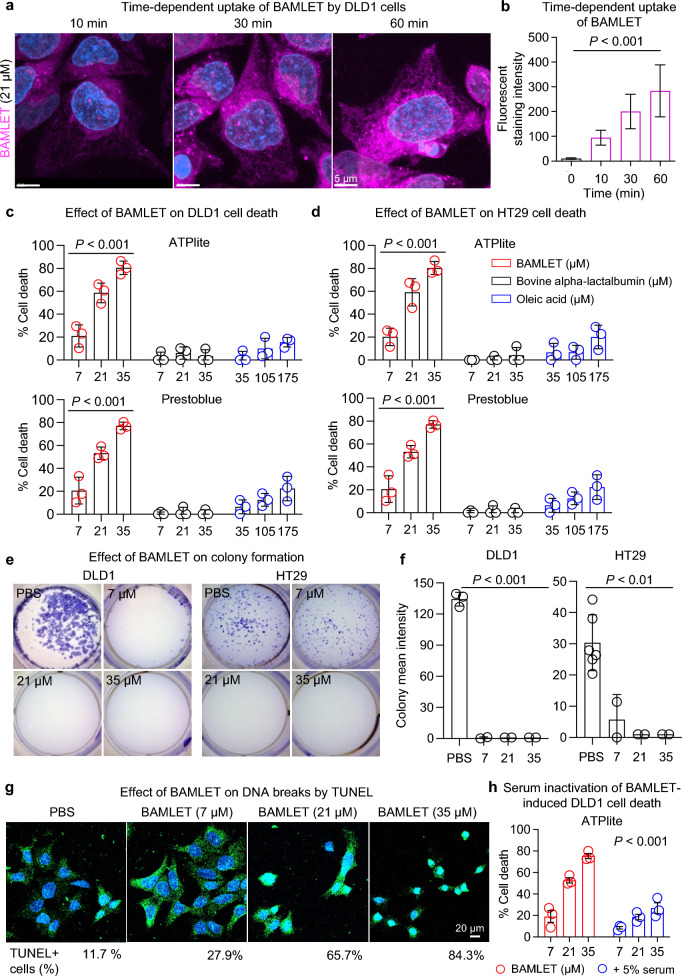
BAMLET interactions with colorectal carcinoma cells. (**a**) Live-cell confocal images showing the time-dependent uptake of Janelia Fluor-549 labeled BAMLET (21 μM, magenta) by DLD1 human colorectal adenocarcinoma cells. Nuclei were counterstained with Hoechst (blue). (**b**) Quantification of the BAMLET cellular uptake in (**a**) (*n* = 50 cells per group). (**c**,**d**) Tumor cell death in BAMLET treated human DLD1 and HT29 colorectal adenocarcinoma cells quantified by ATPlite and Prestoblue assays. Bovine alpha-lactalbumin (black) and oleic acid (blue) did not affect cell viability. (**e**) Tumor cell death quantified by a colony assay, ten days post treatment. DLD1 and HT29 colorectal adenocarcinoma cells were treated with BAMLET or PBS. (**f**) Quantification of colony staining intensity in (**e**). (**g**) Tumor cell apoptosis, defined by a dose-dependent increase in TUNEL staining in BAMLET-treated DLD1 colorectal adenocarcinoma cells. (**h**) Tumor cell death in BAMLET treated human DLD1 colorectal adenocarcinoma cells quantified by ATPlite assay. The addition of 5% serum (FCS) significantly decreased BAMLET-induced cell death. Data are presented as the means ± S.E.M.s from independent experiments, analyzed by two-way ANOVA with Šídák's multiple comparisons test (**c**,**d**,**h**), or two-tailed Kruskal–Wallis test with Dunn’s correction (**b**,**f**). Scale bars = 5 μm (**a**), 20 μm (**g**).

To further define the interaction of BAMLET with tumor cells, colorectal adenocarcinoma cells (DLD1 and HT29) were exposed in vitro to JF549-labeled BAMLET and the interaction of the complex with the cells was followed, by live-cell confocal imaging (Fig. 7a,b). DLD1 cells rapidly internalized BAMLET and a time-dependent increase in cellular BAMLET content was detected in the cytoplasm and perinuclear area. The complex was further shown to accumulate in the nuclei of the treated carcinoma cells. Parallel analysis of cells treated with JF549-labeled bovine alpha-lactalbumin, showed significant uptake, which occurred more slowly and to a lower extent than for the complex (Supplementary Fig. S10).

The cellular uptake of BAMLET was accompanied by cell death, as shown by a dose-dependent reduction in ATP levels and increase in Prestoblue levels, compared to the untreated cells (Fig. 7c,d). In contrast, the bovine alpha-lactalbumin protein or oleic acid alone did not significantly affect cell viability (Fig. 7c,d). A lasting loss of proliferative capacity was demonstrated using the colony assay, in which surviving cells were quantified by colony formation after ten days. The colony number was significantly reduced in the BAMLET-treated cells (Fig. 7e,f). The concentrations of BAMLET (7, 14 and 21 µM) were selected based on extensive previous in vitro studies of HAMLET and BAMLET induced cell death with a three-hour protocol^25,28,29^. No tumoricidal effects were observed in cells exposed to 1, 3 or 5 µM of BAMLET.

BAMLET induced cell death further showed characteristics of apoptosis, defined by DNA double-strand breaks, detected by TUNEL staining. A dose-dependent increase in the frequency of apoptotic cell nuclei was observed. Effects on the chromatin structure of cancer cells have previously been reported for HAMLET and alpha1-oleate^18,30^ (Fig. 7g). The HAMLET and BAMLET complexes are inactivated in serum, due to a higher affinity of serum albumin for oleic acid, compared to alpha-lactalbumin, and the resulting dissociation of the complex^22^. BAMLET-induced cell death was inhibited by serum, as shown by comparing DLD1 cells exposed to BAMLET in the presence or absence of serum (Fig. 7h). This observation makes it less likely that circulating BAMLET complexes account for the protection of extra-intestinal tissues.

The rapid uptake of BAMLET by colorectal adenocarcinoma cells provides a mechanism for the efficient uptake and retention of BAMLET in tumor tissue, detected by whole-intestine imaging and immunohistochemistry.

### Effects of BAMLET on intestinal tissues in healthy C57BL/6 mice

**Figure 8:**
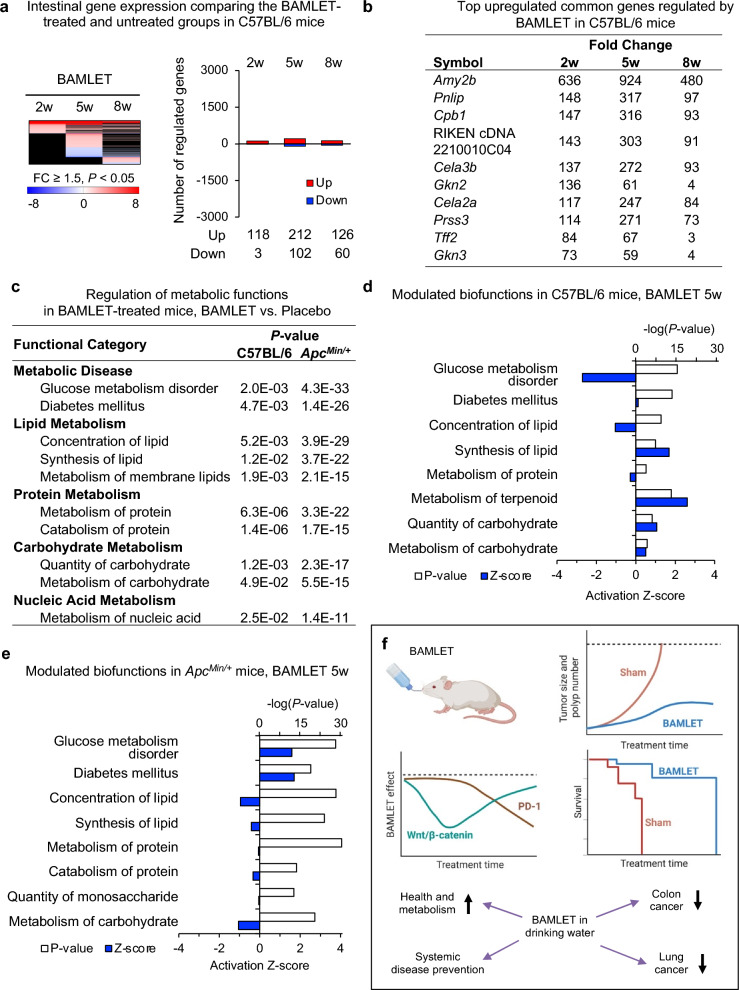
Effects of BAMLET on gene expression in the healthy C57BL/6 mice compared to the tumor prone *Apc*^*Min/*+^ mice. A schematic of the protocol is shown in Fig. 1b. BAMLET (20 mg in 2 × 200 μl PBS daily for ten days) or PBS was administered to *Apc*^*Min/*+^ mice or healthy C57BL/6 mice by gavage (*n* = 5 mice per group) and intestinal tissue segments were obtained two (2w), five (5w) weeks after the end of treatment. BAMLET was further administered in the drinking water for eight weeks (8w). Intestinal tissues were subjected to gene expression analysis (*n* = 1–2 RNA samples per group). (**a)** Heatmap comparing intestinal gene expression profiles in the mice receiving BAMLET to healthy, untreated C57BL/6 mice (red: upregulated genes, blue: downregulated genes, black: nonregulated genes, cutoff FC ≥ 1.5, *P* < 0.05, compared to the untreated group, *n* = 2 RNA samples per group). The total number of regulated genes was low in the healthy C57BL/6 mice (approximately 150 genes), with no evidence of a toxic response to BAMLET. (**b**) The top regulated common genes in the BAMLET-treated C57BL/6 mice were mostly enzymes related to carbohydrate metabolism, lipid metabolism and protein degradation (**c**) Metabolic function analysis of the genes regulated in *Apc*^*Min/*+^ mice at all time points predicted effects on lipid metabolism, glucose metabolism, insulin tolerance and inflammation. A weak regulation was observed in C57BL/6 mice treated with BAMLET, compared to *Apc*^*Min/*+^ mice. (**d**) Enrichment *P*-values and activation Z-scores of biofunctions regulated in the BAMLET-treated *Apc*^*Min/*+^ mice (5w post treatment). (**e**) Enrichment *P*-values and activation Z- scores of biofunctions regulated in the BAMLET-treated C57BL/6 mice (5w post treatment). (f**)** Schematic of the BAMLET effects discussed in this study.

To investigate if BAMLET treatment affects intestinal tissues in healthy mice, C57BL/6 mice were administered BAMLET perorally for ten days and followed post treatment for two or five weeks. A third group was administered BAMLET in drinking water for eight weeks, according to the protocol in Fig. 1. The intestines were examined using the same parameters as in the *Apc*^*Min/*+^ mice (Fig. 8). There was no change in body weight, no evidence of macroscopic changes in intestinal tissue or extra-intestinal organs (Supplementary Fig. S11). A lack of change by histopathology, suggested that BAMLET in the drinking water does not cause adverse effects in healthy mice. This conclusion was supported by gene expression analysis, which did not detect effects on toxicity-associated end points such as cell death, inflammation, or immunity (Fig. 8).

While very few genes were regulated by BAMLET in C57BL/6 mice (*n* = 121–314), gene expression analysis detected unexpected, potentially beneficial effects on metabolism. The analysis was therefore refocused on metabolic endpoints, comparing healthy C57BL/6 mice to *Apc*^*Min/*+^ mice. Metabolic functions were regulated in healthy C57BL/6 mice (Fig. 8a–d). Top upregulated genes included amylases that are important for carbohydrate digestion (*Amy2b*)*,* lipases for lipid digestion (*Pnlip*) and several proteases involved in protein digestion (*Cpb1, Prss3*, *Cela3b*, *Cele2a*), suggesting accelerated or enhanced digestion.

The metabolic response was significantly higher in *Apc*^*Min/*+^ mice treated with BAMLET compared to the placebo-treated mice (Fig. 8c). BAMLET treatment was shown to affect lipid and carbohydrate metabolism in *Apc*^*Min/*+^ mice, compared to placebo (Fig. 8d). Genes regulating glucose and carbohydrate metabolism, lipid metabolism and protein metabolism were significantly inhibited (Supplementary Fig. S12). The HIF1α pathway was strongly inhibited by BAMLET in the *Apc*^*Min/*+^ mice compared to placebo, including genes involved in ATP synthesis, glucose metabolism and cell survival (Supplementary Fig. S12). Peroxisome proliferator-activated receptor (PPAR) signaling pathway was inhibited, suggesting effects on lipid metabolism (Supplementary Fig. S12). PPAR is a nuclear transcription factors that responds to dietary stimuli including fatty acids. Additional regulated transcription factors, known to be involved in metabolism included Ras and NF-κB, affecting the expression of genes such as *Hras, Rala, Hk1, Mapk3, Chuk, Nfkb1* and *Rela,* which were inhibited*.*

This gene expression data is suggestive of a beneficial anti-cancer effect through regulation of lipid and glucose metabolisms, previously demonstrated by metabolomics analysis in tumor cells, exposed to the HAMLET complex, where glucose and lipid deprivation sensitized tumor cells to HAMLET-induced cell death^31^. The results further suggest that BAMLET serves as a tumor surveillance molecule with potent anti-tumor effects in the intestine, executed by the removal of tumor cells and reprogramming of gene expression in affected tissues. In addition, BAMLET reduced Wnt/$\upbeta$-catenin expression in multiple extra-intestinal organs, suggesting protection against Wnt/$\upbeta$-catenin driven tumor development. These effects were largely specific for tumor-bearing mice, as healthy mice treated with BAMLET showed mild regulation of key metabolic functions (Fig. 8f).

## Discussion

This study examined the potential of BAMLET as a peroral therapeutic tool against intestinal cancer in *Apc*^*Min/*+^ mice, an animal model of intestinal cancer. BAMLET was retained in tumor tissue for at least 48 h after the first oral dose. Gavage treatment with BAMLET for ten days was sufficient to inhibit tumor development, and the administration of BAMLET via the drinking water for eight weeks prevented tumor development and inhibited tumor gene expression, attenuating disease severity and resulting in a near healthy phenotype. Prolonged BAMLET treatment further delayed tumor development and increased the survival of *Apc*^*Min/*+^ mice, suggesting that the therapeutic and prophylactic potential of BAMLET should be further explored.

The molecular basis of these therapeutic effects was investigated by analysis of gene expression and tissue staining. Genes that define the tumor microenvironment were strongly upregulated in the placebo-treated *Apc*^*Min/*+^ mice with established cancer but not expressed or weakly expressed in the BAMLET-treated group at the same time points. Genes that drive metastasis, tumor growth, angiogenesis and the Wnt/β-catenin signaling pathway were strongly affected. While these effects may reflect the delay in tumor development observed in the BAMLET-treated mice, our results suggest that BAMLET actively protects the tissue environment by inhibiting or preventing major, cancer-related gene networks from being expressed. Some of these effects, including inhibition of genes involved in metastasis, tumor growth, angiogenesis and the Wnt/β-catenin signaling, were still detected after long-term follow-up, suggesting that BAMLET administration in the drinking water maintains anti-tumor pressure by removing emergent cancer cells and reprogramming gene expression in intestinal tissues.

Notably, the pronounced anti-tumor effects of BAMLET in *Apc*^*Min/*+^ mice also included extra-intestinal tissues. Genes defining the tumor microenvironment were strongly inhibited by peroral BAMLET treatment, in the lungs, livers and kidneys. Most *Apc*^*Min/*+^ mice die of anemia or intussusception with an expected life span of about 100 days^32^ rather than metastatic disease and *Apc*^*Min/*+^ mice are not used as a model to study colon cancer metastasis. We detected proliferating cell foci in the lungs of the placebo-treated *Apc*^*Min/*+^ mice and staining for the lung adenocarcinoma marker TTF-1^27^ suggested a lung origin rather than metastases from the intestinal tumors. The Wnt/β-catenin pathway is operative in the adult lung epithelium^33^ and, patients with familial adenomatous polyposis have been reported to develop lung cancer^34^, suggesting that aberrant Wnt/β-catenin signaling may drive extra-intestinal tumor development; a process that appeared to be inhibited by BAMLET administration in the drinking water.

BAMLET was shown to also affect metabolic functions, mainly in the tumor prone *Apc*^*Min/*+^ mice and in healthy C57BL/6 mice. In a previous study, a combination of small-hairpin RNA (shRNA) inhibition, proteomic and metabolomic technology, identified the c-Myc and Ras oncogenes, as determinants of sensitivity to HAMLET^31^. HAMLET sensitivity was further modified by the glycolytic state of tumor cells as glucose deprivation sensitized tumor cells to HAMLET-induced cell death and in the shRNA screen, hexokinase 1 (HK1), 6-phosphofructo-2-kinase/fructose-2,6-biphosphatase 1 and HIF1α, modified HAMLET sensitivity^31^. In this study, biofunctions such as obesity, insulin resistance and glucose metabolism were significantly affected by BAMLET in *Apc*^*Min/*+^ mice, which had less severe disease due to the removal of cancer cells and delay in tumor progression. Interestingly, beneficial effects of bovine alpha-lactalbumin treatment were previously detected in obese mice on a high-fat diet, with improved glucose tolerance, adiponectin function and reduced insulin resistance^35–37^. It may be speculated that the presence of fatty acids in the tissues of obese mice may result in spontaneous BAMLET formation with bovine alpha-lactalbumin, which might improve metabolism.

PD-1 protein levels were markedly reduced in the BAMLET-treated *Apc*^*Min/*+^ mice compared to the placebo group and the PD-1 signaling pathway was inhibited compared to that in the placebo group, in which the intestinal PD-1 signaling pathway and PD-1 staining were enhanced, compared to the healthy mice. The functional consequences of this observation are not clear, as the efficacy of immune checkpoint inhibitors, such as anti-PD-1/PD-L1 therapy^38^, is limited for the treatment of colorectal cancer and restricted to the treatment of microsatellite instability-high (MSI-H) tumors^39^. For patients with microsatellite stability (MSS) colorectal cancer, the response rate is only 5–10%^40^. Except for the PD-1-associated human leukocyte antigens (HLA)^41^, which were inhibited, there was no evidence of a more general effect of BAMLET on the immune response in intestinal tissues.

Alpha-lactalbumin acts as a substrate specifier in the lactose synthase complex and the production of lactose is essential for the survival of mammals. Mice engineered not to express alpha-lactalbumin are unable to express milk and feed their offspring^42^. Our results suggest that the ability of alpha-lactalbumins to form oleic acid complexes may provide additional advantage during breast-feeding, through the removal immature intestinal cells or de-differentiating cells that resemble tumor cells. Finally, the effect of BAMLET on metabolic functions in vivo, suggests that complexes formed by alpha-lactalbumin and oleic acid may have broader nurturing effects.

The present study supports the potential of BAMLET as a tumor surveillance molecule for preventive or therapeutic use in cancer-prone patients is supported by this study. We found evidence that intestinal polyp formation was inhibited by administration of BAMLET in the drinking water and long-term protection was achieved in this model, as shown by lower tumor numbers and increased survival. In addition, extra-intestinal disease was prevented by BAMLET treatment. These positive effects and the apparent lack of toxicity of BAMLET suggest the potential of local administration of BAMLET to prevent or treat intestinal cancers and their systemic effects. The structural and functional diversity of alpha-lactalbumin may be essential to maintain health in the intestinal tract and possibly in other organs of the breast-fed infant. It may be speculated that evolution favors proteins like alpha-lactalbumin that successfully adapt their structure to gain functional diversity^9,16^.

## Methods

### Study design

This study addressed if molecules that drive tissue differentiation in the newborn intestine may moonlight as tumor surveillance molecules. The BAMLET complex was selected due to its potent tumoricidal effects against human colon cancer cells and was administered perorally to intestinal cancer prone *Apc*^*Min/*+^ mice. Treatment effects were quantified consecutively by measuring survival, general health parameters, macroscopic tumor growth and by high-resolution tissue imaging. Effects of treatment on the tissue response was analyzed by gene expression and immunohistochemistry, and molecular mechanisms by staining for specific markers associated with cancer progression.

Two peroral BAMLET treatment protocols were used, a short (ten days) treatment by oral gavage to study the therapeutic effects, and a long (eight weeks minimum) treatment in the drinking water to study long-term protection and survival. A strong, protective effect was first documented as a marked difference in the number and size of intestinal tumors in the treated mice compared to the placebo group. Short-term inhibition of tumor development in the BAMLET-treated mice was identified and with extended treatment, protection was prolonged, resulting in a marked increase in survival beyond 250 days compared to about 180 days in the controls. The analysis of macroscopic end points and tissue pathology was extended by gene expression analysis identifying cancer-specific responses and affected tumor markers.

### Preparation of the BAMLET complex

The BAMLET complex was made by mixing bovine alpha-lactalbumin (Agropur, Cat# BiPRO Alpha 9000; Sigma-Aldrich, Cat# L6010) with oleic acid (Sigma-Aldrich, Cat# O1008) in PBS (0.137 M Sodium chloride, 0.0027 M Potassium Chloride, 0.01 M Sodium Phosphate Dibasic, 0.0018 M Potassium Phosphate Monobasic, pH = 7.2–7.4) and vortexing for 30 s. The stoichiometry of protein to oleic acid was 1:5, which has been shown by NMR to result in a mixture where the fatty acid is bound to bovine alpha-lactalbumin, without an excess of free fatty acid. The complex was used immediately for experiments. Bovine alpha-lactalbumin and oleic acid were used as controls. The BAMLET complex has been extensively characterized in previous studies, including the stoichiometry of protein and oleic acid^8,9,43^.

### The intestinal cancer model in ApcMin/+ mice

*Apc*^*Min/*+^ (C57BL/6J-*Apc*^*Min*^/J, RRID:IMSR_JAX002020)^6,7,26^ mice and C57BL/6 were obtained from The Jackson Laboratory at approximately eight weeks of age. *Apc*^*Min/*+^ mice carry a heterozygote mutation in the murine *Apc* (*adenomatous polyposis coli*) locus, encoding a nonsense mutation at codon 850 and resulting in multiple intestinal neoplasia (*Min*). The mice were housed in cages (four to five animals per cage) with food and water ad libitum under a 12:12 light–dark cycle at 22–25 °C. Genotyping was performed by PCR analysis of genomic DNA obtained from blood collected from the retro-orbital sinus. In this mouse model, multiple tumors develop in the small intestine at eight to ten weeks. Mice were acclimated for approximately two weeks at the local animal facility at Biomedical Centre (BMC), Lund University, to reduce stress from transportation.

For the BAMLET therapeutic protocol, ten-week-old female mice (*n* = 10–14 per group) were orally gavaged with 20 mg of BAMLET daily for ten days in 2 × 200 μl of PBS (10 mg/200 μl twice daily). The mice were not given water or food five hours prior to BAMLET administration. Food and water were provided 30 min after oral administration of BAMLET. The placebo-treated mice were gavaged with 2 × 200 μl of PBS daily for ten days. Mice were sacrificed by isoflurane anesthesia overdose (Dechra, Cat# 200–129) two weeks and five weeks after the end of the treatment, and intestinal tissue samples were collected for further analysis.

For the BAMLET long-term treatment protocol, ten-week-old female *Apc*^*Min/*+^ mice (*n* = 10–15 mice per group) were provided daily with BAMLET (20 mg/day in 5 ml of PBS) in the drinking water for eight weeks and sacrificed at 37 weeks of age. The placebo-treated mice received the PBS vehicle as drinking water. The same treatment protocol was used in the survival study groups, in which mice were observed until 37 weeks of age. Mice were inspected daily for health issues and deaths and euthanasia was performed only on those mice that showed one or more of the major signs: weight loss of > 20%, hunched back, prostration, low response to the stimulation and lack of grooming.

### Tumor enumeration and sample collection

Mice were sacrificed by isoflurane inhalation. Tumor enumeration and sample collection were performed as previously described^24^. Tumor numbers and sizes were determined using a dissecting microscope (Olympus). Briefly, the number of polyps was counted in the entire small intestine of each animal. Counting was blinded as to the origin of the tumors and experimental group. For further analysis, the intestines were divided into three parts from the top. The first part was taken for RNA analysis. The second part was used for Swiss roll preparations followed by fixation and staining. The third part was used for methylene blue staining. Tumor numbers, gene expression data, pathology and tissue staining were performed for whole tissue samples, without any selection of areas with or without tumor.

### Methylene blue staining

The opened intestinal segments were spread flat between sheets of filter paper and fixed overnight in 10% neutral buffered formalin. Formalin-fixed sections were transferred to 70% ethanol and stained with 0.2% methylene blue (Sigma-Aldrich, Cat# M9140). The stained sections were rinsed in deionized water and imaged by a dissecting microscope.

### Histology and immunohistochemistry

Small intestines were cut open longitudinally, the intestinal contents were carefully removed, and each intestine divided into three segments. One segment was rolled up using a wooden stick to form a Swiss roll and was fixed overnight in 10% neutral buffered formalin. Samples were embedded in paraffin, and 5-µm-thick sections were further processed for histology and immunohistochemistry using antibodies for bovine alpha-lactalbumin (Thermo Fisher Scientific, Cat# A10-128A, 1:250), β-catenin (Cell Signaling, Cat# 9562, 1:250), cyclin D1 (Thermo Fisher Scientific, Cat# SC8396, 1:250), Ki67 (BD Biosciences, Cat# 556003, 1:200), VEGF (Abcam, Cat# ab46154, 1:200) and TTF-1 (Abcam, Cat# ab227652, 1:250) as previously described^24^ with slight modifications. Citrate buffer (Dako Target Retrieval Solution, Agilent, Cat# S1699) was used for antigen retrieval for cyclin D1, Ki67, VEGF, bovine alpha-lactalbumin and β-catenin staining. EDTA buffer (Abcam, Cat# ab64216) was used for antigen retrieval for TTF-1 staining. Immunohistochemistry was quantified by ImageJ. Hematoxylin (Thermo Fisher Scientific, Cat# 7211) and eosin (Thermo Fisher Scientific, Cat# 7111) staining was used to identify histopathological changes in all of the organs and were carried out according to the manufacturer’s instruction.

Images were captured using the AX10 microscope (Carl Zeiss) or a Nanozoomer scanner (Hamamatsu), and the number of diaminobenzidine (DAB)-positive fields (4–8) in regions of healthy and tumor tissue were identified in the cross-sections of each organ. Image J’s immunohistochemistry profiler was used for color deconvolution, by which DAB brown stain was separated from the Mayer’s hematoxylin counterstain. ImageJ inverts the images and after inversion, the DAB-stained areas become bright, and unstained areas are dark. Staining of specific areas within individual tissues was compared by analysis of areas where the changes occurred to the same number of areas where the changes did not occur.

### Immunofluorescence staining

For PD-1 immunofluorescence staining, paraffin sections were deparaffinized in xylene, rehydrated with a decreasing series of ethanol concentrations and then washed with deionized water. The slides were then immersed in citrate buffer (Dako Target Retrieval Solution, Agilent, Cat# S1699) and boiled for 20 min, followed by 30 min of permeabilization with 0.25% Triton in PBS at room temperature. The sections were incubated with a blocking solution containing 5% goat serum in PBS for one hour at room temperature before the anti-rabbit PD-1 antibody (Abcam, Cat# ab214421, 1:150) was added and incubated overnight at 4 °C. The slides were then washed with 0.025% PBS-Tween and stained with goat anti-rabbit Alexa Fluor-568 secondary antibody (Thermo Fisher Scientific, Cat# A11034, 1:200) for one hour at room temperature. The nuclei were counterstained with DAPI for 15 min, washed in PBS, and then mounted with Fluoromount aqueous mounting medium (Sigma-Aldrich, Cat# F4680). Images were captured with a Hamamatsu Nanozoomer scanner, and the fluorescence intensity in regions of healthy and tumor tissues in the cross-sections was quantified by ImageJ. After splitting the different color channels, PD-1 fluorescence intensity (magenta color channel) was quantified in tumor areas and healthy areas in each section, and the placebo- and BAMLET-treated groups were compared.

### Real-time in vivo fluorescence imaging of BAMLET

BAMLET was labeled using a VivoTag 680XL Protein Labeling Kit (Perkin Elmer). *Apc*^*Min/*+^ mice were orally gavaged with 10 mg of VivoTag 680-labeled BAMLET in 200 μl of PBS. The intestinal tissue samples were collected after 24 h or 48 h and imaged using an IVIS Spectrum imaging system (Perkin Elmer). The BAMLET fluorescent signal was acquired at an excitation wavelength of 680 nm.

### Transcriptomic analysis

Approximately 5 mg of tissue was homogenized using a Tissuelyser (Qiagen), and total RNA was extracted using the RNeasy kit (Qiagen) with on-column DNA digestion. Exactly 100 ng of total RNA was amplified and fragmented using the Affymetrix WT PLUS Reagent or GeneChip 3´IVT Express kits, followed by hybridization onto Clariom S Mouse HT or Mouse Genome 430 PM arrays (all Affymetrix) for 16 h at 45 °C, washed, stained (Applied Biosystems, ThermoFisher Scientific) and scanned using the GeneTitan or GeneAtlas system. Data were normalized using Robust Multi Average implemented in the Transcriptome Analysis Console software (v.4.0.1.36, Applied Biosystems, Thermo Fisher Scientific). Relative expression was analyzed by ANOVA using the empirical Bayes method, and genes with an absolute FC ≥ 1.5 or 2.0 and *P*-value < 0.05 were considered differentially expressed. Heatmaps were constructed using GraphPad Prism 9.

Differentially expressed genes and regulated pathways were analyzed using the Core Analysis feature of Ingenuity Pathway Analysis software^44^ (IPA, Qiagen), using right-tailed Fisher's Exact Test followed by Benjamini–Hochberg correction for multiple testing. Canonical pathways analysis identified the pathways from the QIAGEN Ingenuity Pathway Analysis library of canonical pathways that were most significant to the data sets. Molecules from the data set with FC ≥ 1.5 or 2.0 and adjusted *P-*value < 0.05 that were associated with a canonical pathway in the QIAGEN Knowledge Base were considered for the analysis. The significance of the association was measured using a right-tailed Fisher’s Exact test, where *P*-values determine the probability that the association between the genes in the dataset and the canonical pathway would be explained by chance alone. In addition, a Z-score was calculated to indicate the likelihood of activation or inhibition of each pathway.

### Cell culture

Colorectal adenocarcinoma cells (DLD1, Cat# CCL-221, RRID:CVCL_0248) and colorectal adenocarcinoma cells (HT29, Cat# HTB-38, RRID:CVCL_0320) were purchased from American Type Culture Collection (ATCC). DLD1 and HT29 cells were cultured in RPMI-1640 medium with 1% nonessential amino acids, 1 mM sodium pyruvate, 50 μg/ml gentamicin and 5–10% fetal calf serum (FCS) at 37 °C and 5% CO_2_. All cell culture reagents were purchased from Thermo Fisher Scientific. Cells were subcultured every three days.

### Cell death assays

Two assays were used as indirect measures of cell death: the luminescence-based ATPlite kit (Perkin Elmer, Cat# 6016947) and the Prestoblue cell viability assay (Thermo Fisher Scientific, Cat# A13262). Cells (5 × 10^4^ cells/well) were seeded in serum-free RPMI-1640 medium on 96-well plates, treated with BAMLET, bovine alpha-lactalbumin or oleic acid at different concentrations (7, 21, and 35 µM) and incubated for one hour. Afterward, FCS was added at a concentration of 5%, and the cells were continuously incubated for two hours at 37 °C, at which time the two assays were carried out according to the manufacturers’ instructions. For the serum inhibition assay, DLD1 cells were treated with BAMLET (7, 21, and 35 µM) in the presence of 5% FCS and incubated for three hours at 37 °C. Luminescence and fluorescence were measured using a microplate reader (Infinite F200, Tecan). The experiments were performed in triplicate and repeated trice.

### Colony assay

Cells were seeded on 12-well plates (1 × 10^3^ cells/well) and incubated overnight. Cells were treated with BAMLET (7, 21, and 35 µM) in serum-free medium and incubated for one hour at 37 °C and 5% CO_2_. The incubation was continued after the addition of FCS to the medium. On day ten post-treatment, the cells were washed once and fixed with cold methanol (300 µl) for 15 min on ice. Finally, cell colonies were stained with hematoxylin (Thermo Fisher Scientific, Cat# 7211) for five minutes, and images were captured under a dissecting microscope (Carl Zeiss). The experiment was repeated twice for each cell line.

### Live-cell imaging assay

To visualize the cellular uptake of BAMLET, cells were seeded on 6-well ibidi chambers (3.5 × 10^4^ cells/well) overnight and then treated with Janelia Fluor-549 labeled (Tocris, Cat# 6147) BAMLET mixed 1:1 with unlabeled BAMLET (21 µM) or bovine alpha-lactalbumin (21 µM) for one hour at 37 °C. The nuclei were counterstained for five minutes with Hoechst (1:2,000, Molecular Probes, Cat# H1399), and the uptake of labeled BAMLET was captured with an LSM 900 laser scanning confocal microscope with oil immersion × 63 objectives (Carl Zeiss).

### TUNEL assay

DNA fragmentation was detected using the terminal deoxynucleotidyl transferase dUTP nick end-labeling (TUNEL) assay (Click-iT TUNEL Alexa Fluor 488 imaging assay kit, Thermo Fisher Scientific, Cat# C10245). DLD1 cells were seeded in 8-well chamber slides (2 × 10^4^ cells/well), cultured overnight (37 °C, 5% CO_2_) and incubated with 7, 21 or 35 µM BAMLET for one hour in serum-free RPMI-1640 medium at 37 °C. Cells were fixed (2% PFA, 15 min), permeabilized (0.25% Triton X-100 in PBS, 20 min) and incubated with TUNEL reaction mixture containing TdT for 60 min at 37 °C. After the TUNEL reaction, the cells were incubated with Click-iT reaction mixture for 30 min. Cells were counterstained with Hoechst 33,342 (1:1,000, 15 min, Thermo Fisher Scientific, Cat# 62,249), mounted in Fluoromount aqueous mounting medium (Sigma-Aldrich, Cat# 4680), and examined with an LSM 900 confocal microscope (Carl Zeiss). Fluorescence intensities were quantified by ImageJ.

### Statistical analysis

All in vitro experiments were repeated at least two times. Data were expressed as the means ± S.E.M.s or medians. The normality of the data distribution was determined by the D'Agostino-Pearson normality test. For data following a Gaussian distribution, Student’s *t* test was used. Other data sets were analyzed by the Mann–Whitney U test. Differences between the control and treatment groups were determined by one-way or two-way ANOVA with Šídák’s multiple comparisons test (parametric data) and two-tailed Kruskal–Wallis test with Dunn’s correction (non-parametric data). Significance was assigned at *P* < 0*.*05 and is indicated as follows:* P* < 0*.*05*, P* < 0*.*01 and *P* < 0*.*001*.* Differences in survival were evaluated by Kaplan–Meier analysis with a log-rank (Mantel‒Cox) test.

### Ethical approval

Experiments were approved by the Malmö/Lund Animal Experimental Ethics Committee at the Lund District Court, Sweden (#01302-20). Animal care and animal experimental protocols followed institutional, national, and European Union guidelines and were governed by the European Parliament and Council Directive (2016/63, EU), the Swedish Animal Welfare Act (Djurskyddslagen 1988:534), the Swedish Welfare Ordinance (Djurskydssförordningen 1988:539) and Institutional Animal Care and Use Committee (IACUC) Guidelines. Results were reported in accordance with ARRIVE guidelines (https://arriveguidelines.org).

### Supplementary Information

Supplementary Information 1.

Supplementary Information 2.

## Data Availability

The data supporting the animal and cellular findings of this study are available within the article and its supplementary information files. The microarray data for this study has been deposited in the NCBI’s Gene Expression Ominibus repository under accession number GSE235767.
