# Supplementary material for: BAMLET administration via drinking water inhibits intestinal tumor development and promotes long-term health
Source: Sci Rep. 2024 Feb 15;14:3838. doi: 10.1038/s41598-024-54040-w (PMC10869698; doi:10.1038/s41598-024-54040-w)

Supplementary Figures

Fig. S1

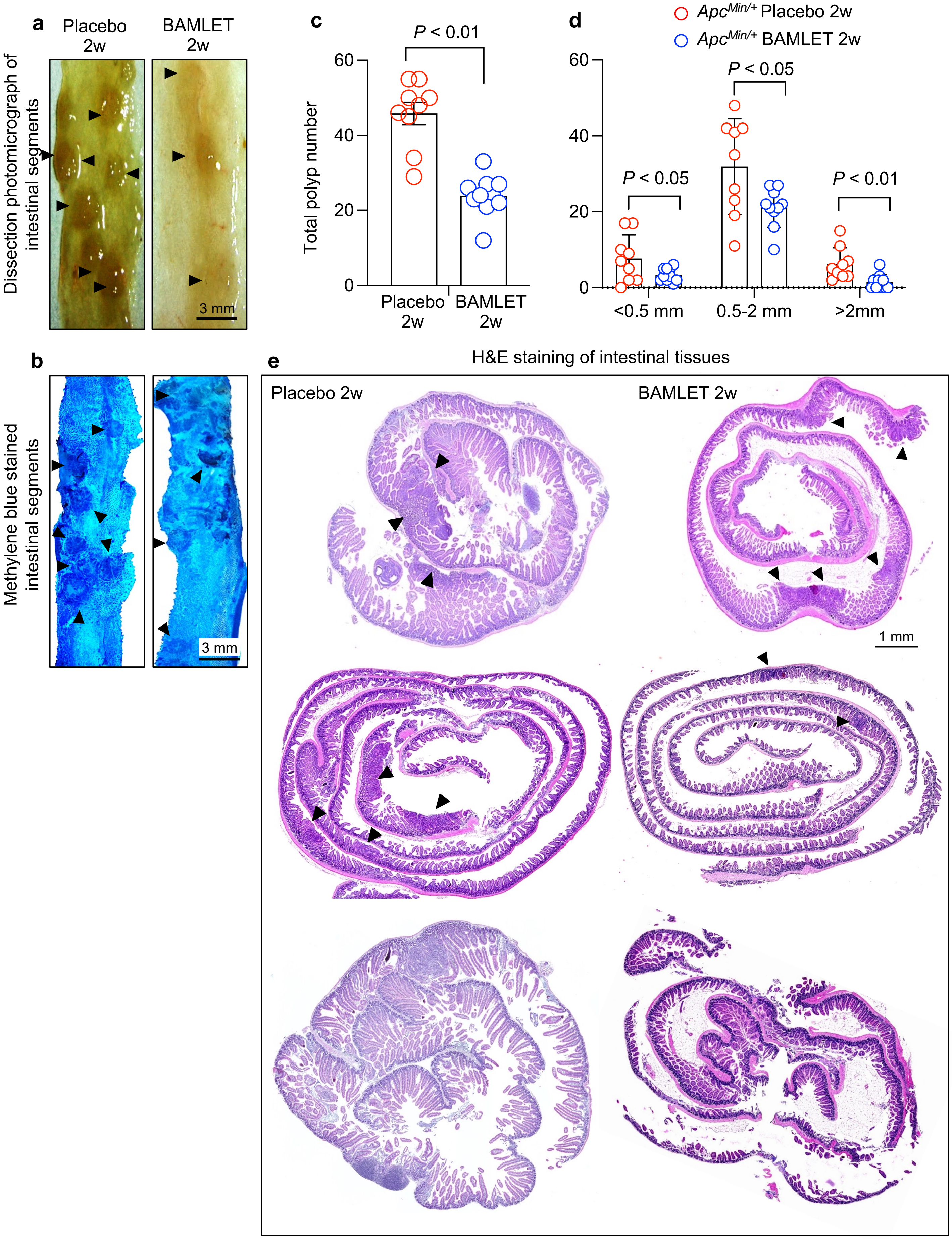

a
Placebo 2w
BAMLET 2w
Dissection photomicrograph of intestinal segments
3 mm
b
Methylene blue stained intestinal segments
3 mm
c
P < 0.01
Total polyp number
60
40
20
0
Placebo 2w
BAMLET 2w
d
ApcMin/+ Placebo 2w
ApcMin/+ BAMLET 2w
60
40
20
0
P < 0.05
P < 0.05
P < 0.01
<0.5 mm
0.5-2 mm
>2mm
e
H&E staining of intestinal tissues
Placebo 2w
BAMLET 2w
1 mm

Fig. S1

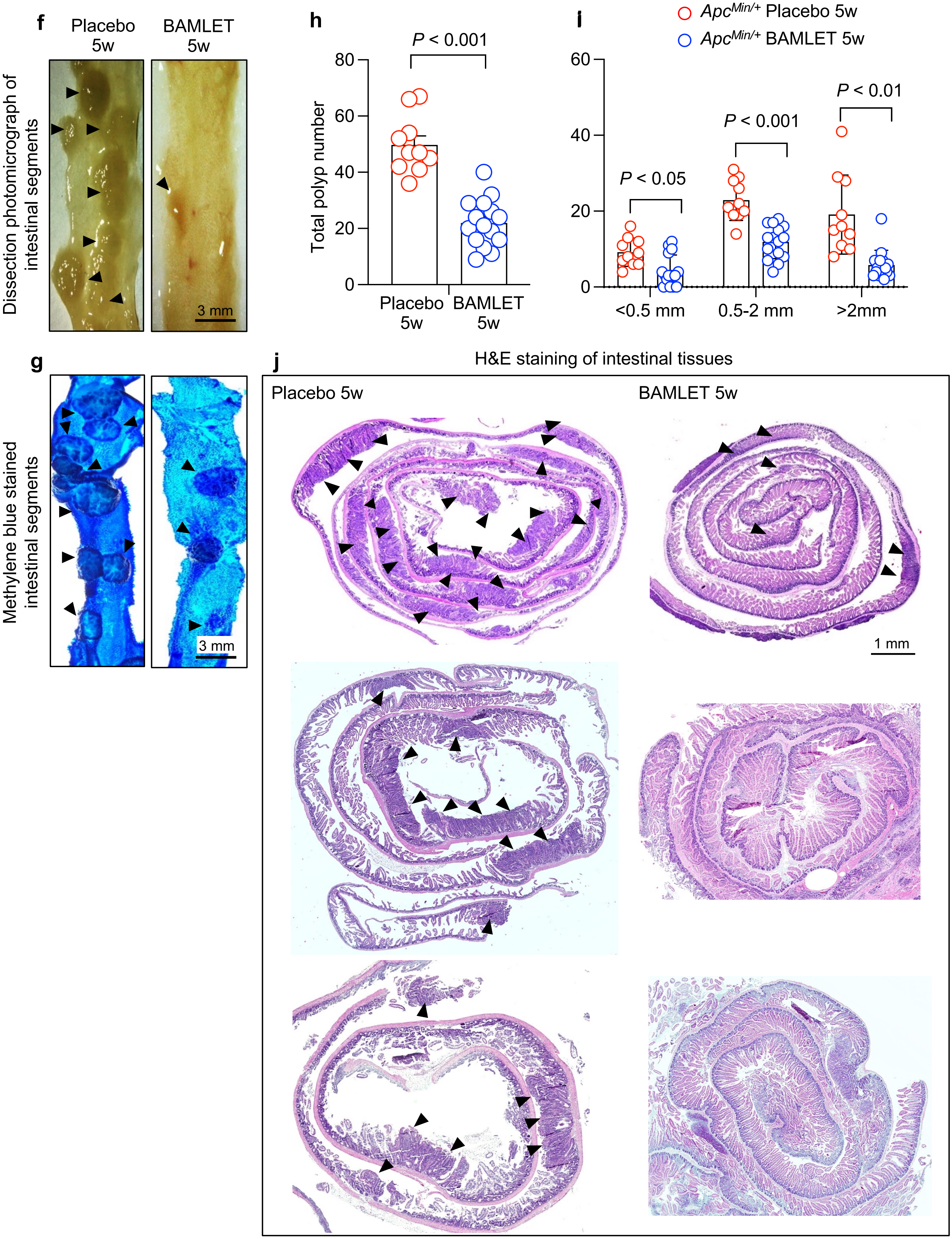
f
Placebo 5w
BAMLET 5w
Dissection photomicrograph of intestinal segments
3 mm
h
P < 0.001
Total polyp number
80
60
40
20
0
Placebo 5w
BAMLET 5w
i
ApcMin/+ Placebo 5w
ApcMin/+ BAMLET 5w
60
40
20
0
P < 0.05
P < 0.001
P < 0.01
<0.5 mm
0.5-2 mm
>2mm
g
Methylene blue stained intestinal segments
3 mm
j
H&E staining of intestinal tissues
Placebo 5w
BAMLET 5w
1 mm

Fig. S1

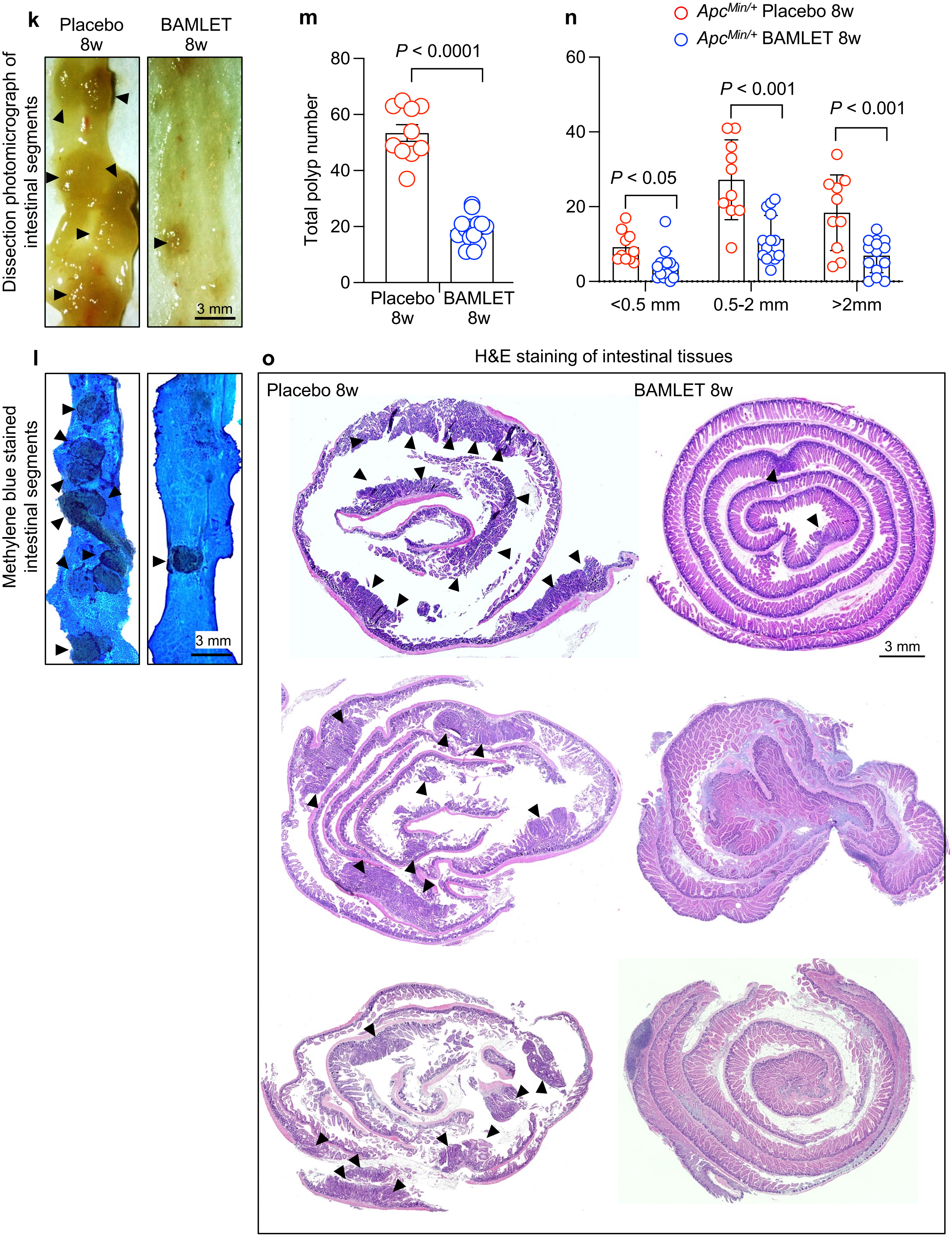

k
Placebo 8w
BAMLET 8w
Dissection photomicrograph of intestinal segments
3 mm
m
P < 0.0001
Total polyp number
Placebo 8w
BAMLET 8w
n
ApcMin/+ Placebo 8w
ApcMin/+ BAMLET 8w
P < 0.05
P < 0.001
P < 0.001
<0.5 mm
0.5-2 mm
>2mm
l
Methylene blue stained intestinal segments
3 mm
o
H&E staining of intestinal tissues
Placebo 8w
BAMLET 8w
3 mm

Fig. S2

Colon cancer related gene expression in the BAMLET-treated and placebo groups in *Apc*$^{Min/+}$ mice

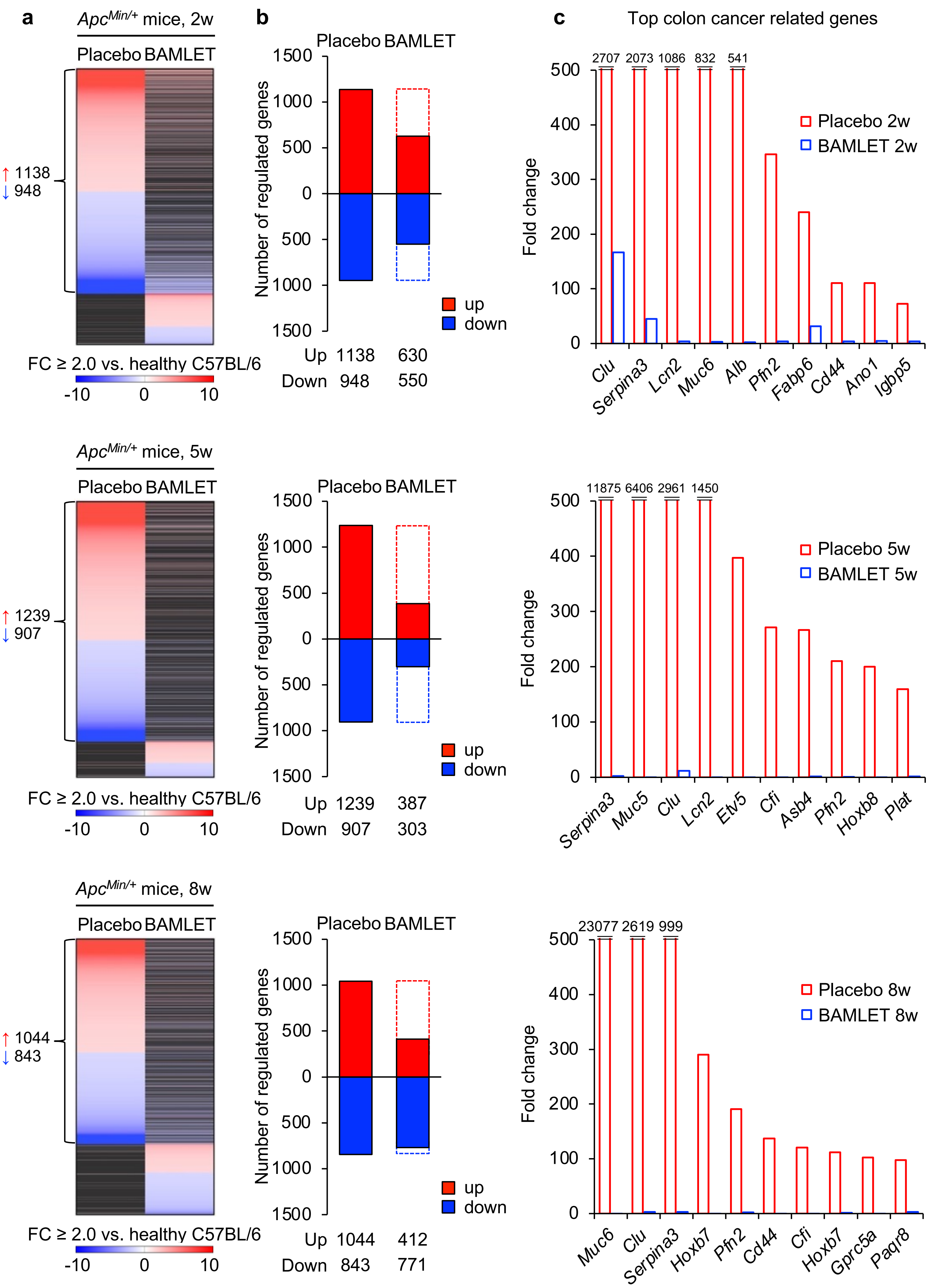

Fig. S3

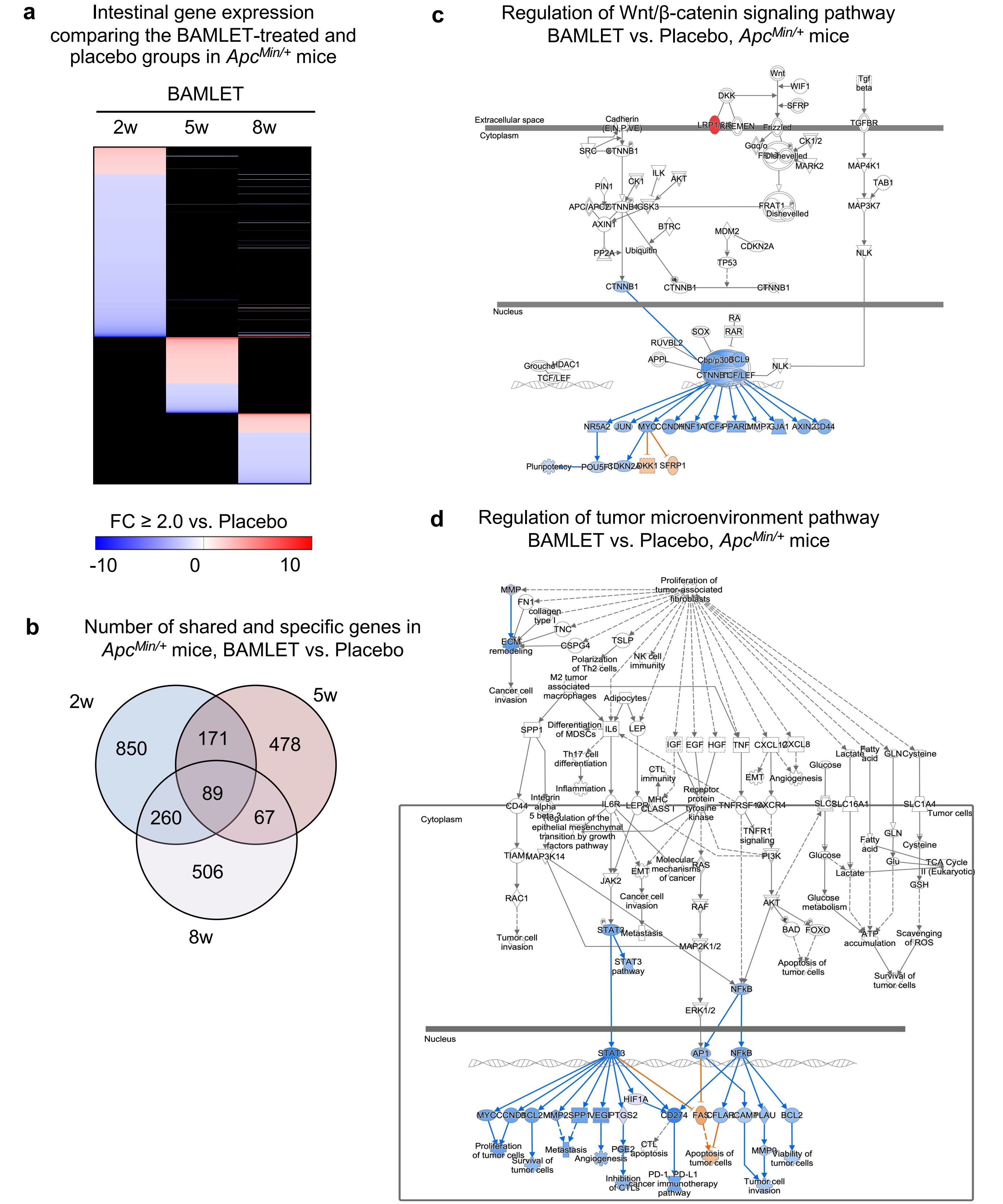

a
Intestinal gene expression comparing the BAMLET-treated and placebo groups in ApcMin/+ mice
BAMLET
2w
5w
8w
FC ≥ 2.0 vs. Placebo
-10
0
10
b
Number of shared and specific genes in ApcMin/+ mice, BAMLET vs. Placebo
2w
5w
8w
850
171
478
89
260
67
506
c
Regulation of Wnt/β-catenin signaling pathway BAMLET vs. Placebo, ApcMin/+ mice
Extracellular space
Cytoplasm
Nucleus
Wnt
WIF1
DKK
SFRP
Tgf beta
TGFBR
Cadherin (E,N,P,VE)
LRP1/5/6
KREMEN
Frizzled
SRC
CTNNB1
Gαq/o
CK1/2
Dishevelled
MARK2
MAP4K1
TAB1
MAP3K7
NLK
PIN1
CK1
ILK
AKT
APC/APC2
GSK3
FRAT1
AXIN1
BTRC
MDM2
CDKN2A
TP53
PP2A
Ubiquitin
RA
RAR
SOX
RUVBL2
APPL
Groucho
HDAC1
TCF/LEF
Cbp/p300
BCL9
NR5A2
JUN
MYC
CCND1
HNF1A
TCF4
PPARD
MMP7
GJA1
AXIN2
CD44
Pluripotency
POU5F1
CDKN2A
DKK1
SFRP1
d
Regulation of tumor microenvironment pathway BAMLET vs. Placebo, ApcMin/+ mice
Proliferation of tumor-associated fibroblasts
MMP
FN1
collagen type I
TNC
ECM remodeling
CSPG4
TSLP
Polarization of Th2 cells
NK cell immunity
M2 tumor associated macrophages
Cancer cell invasion
Adipocytes
SPP1
Differentiation of MDSCs
IL6
LEP
IGF
EGF
HGF
TNF
CXCL12
CXCL8
Lactate
Fatty acid
GLN
Cysteine
Th17 cell differentiation
CTL immunity
Glucose
EMT
Angiogenesis
Inflammation
Receptor protein tyrosine kinase
Cytoplasm
CD44
Integrin alpha 5 beta 3
IL6R
LEPR
MHC CLASS I
TNFRSF1A
CXCR4
SLC5
SLC16A1
SLC1A4
Tumor cells
Regulation of the epithelial mesenchymal transition by growth factors pathway
TNFR1 signaling
GLN
Fatty acid
Cysteine
TIAM
MAP3K14
PI3K
Glucose
Glu
TCA Cycle II (Eukaryotic)
Lactate
Molecular mechanisms of cancer
EMT
RAS
GSH
JAK2
RAC1
Cancer cell invasion
AKT
Glucose metabolism
RAF
STAT3
Metastasis
BAD
FOXO
ATP accumulation
Scavenging of ROS
Tumor cell invasion
MAP2K1/2
STAT3 pathway
Apoptosis of tumor cells
NFkB
Survival of tumor cells
ERK1/2
Nucleus
STAT3
AP1
NFkB
HIF1A
MYC
CCND1
BCL2
MMP2
SPP1
VEGF
PTGS2
CD274
FAS
CFLAR
ICAM1
PLAU
BCL2
Proliferation of tumor cells
Survival of tumor cells
Metastasis
PGE2
Angiogenesis
CTL apoptosis
Apoptosis of tumor cells
MMP9
Viability of tumor cells
Inhibition of CTLs
PD-1, PD-L1 cancer immunotherapy pathway
Tumor cell invasion

Fig. S4

Effect of BAMLET treatment on tumor markers in *Apc*$^{Min/+}$ mice

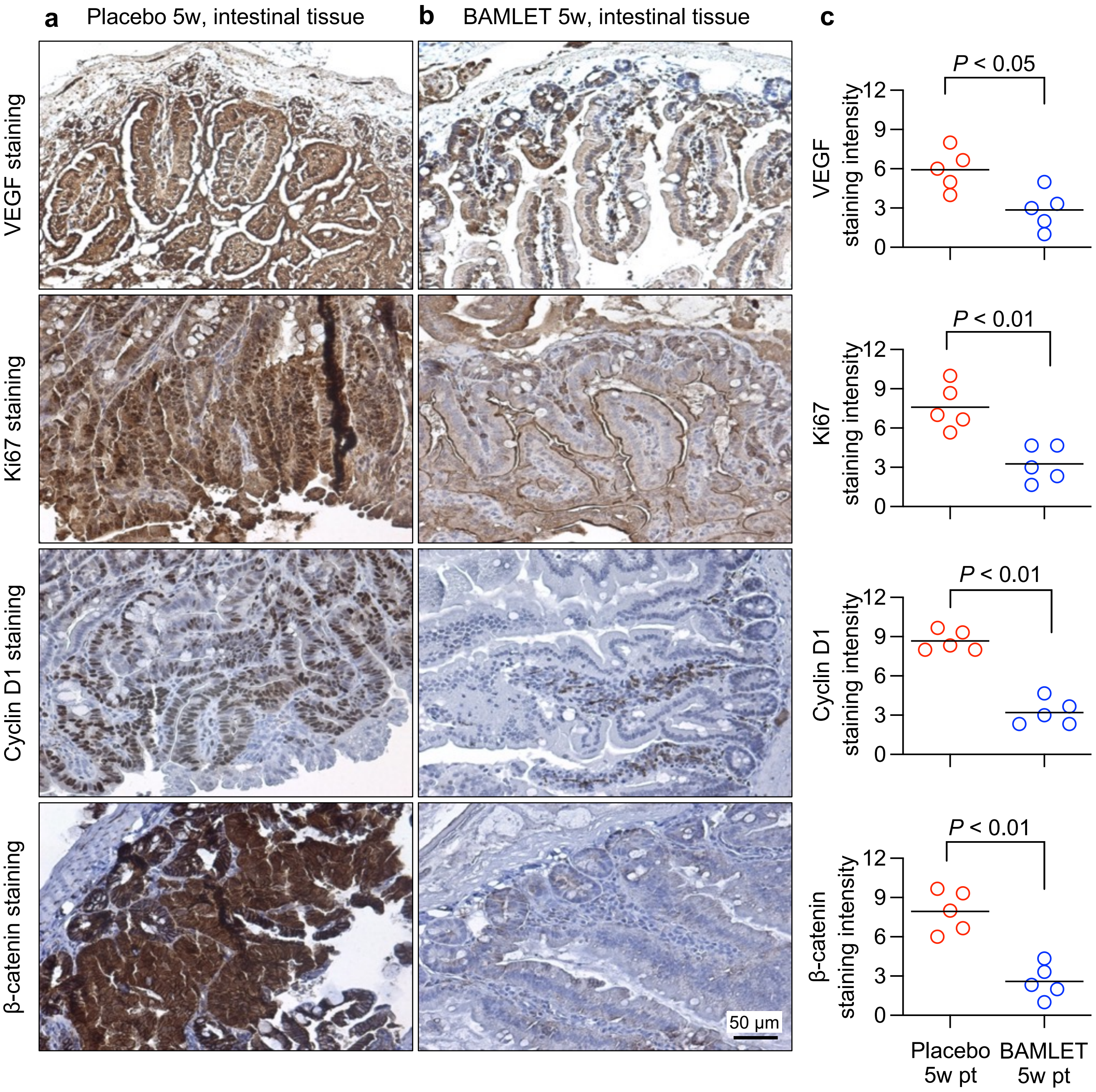

Fig. S4

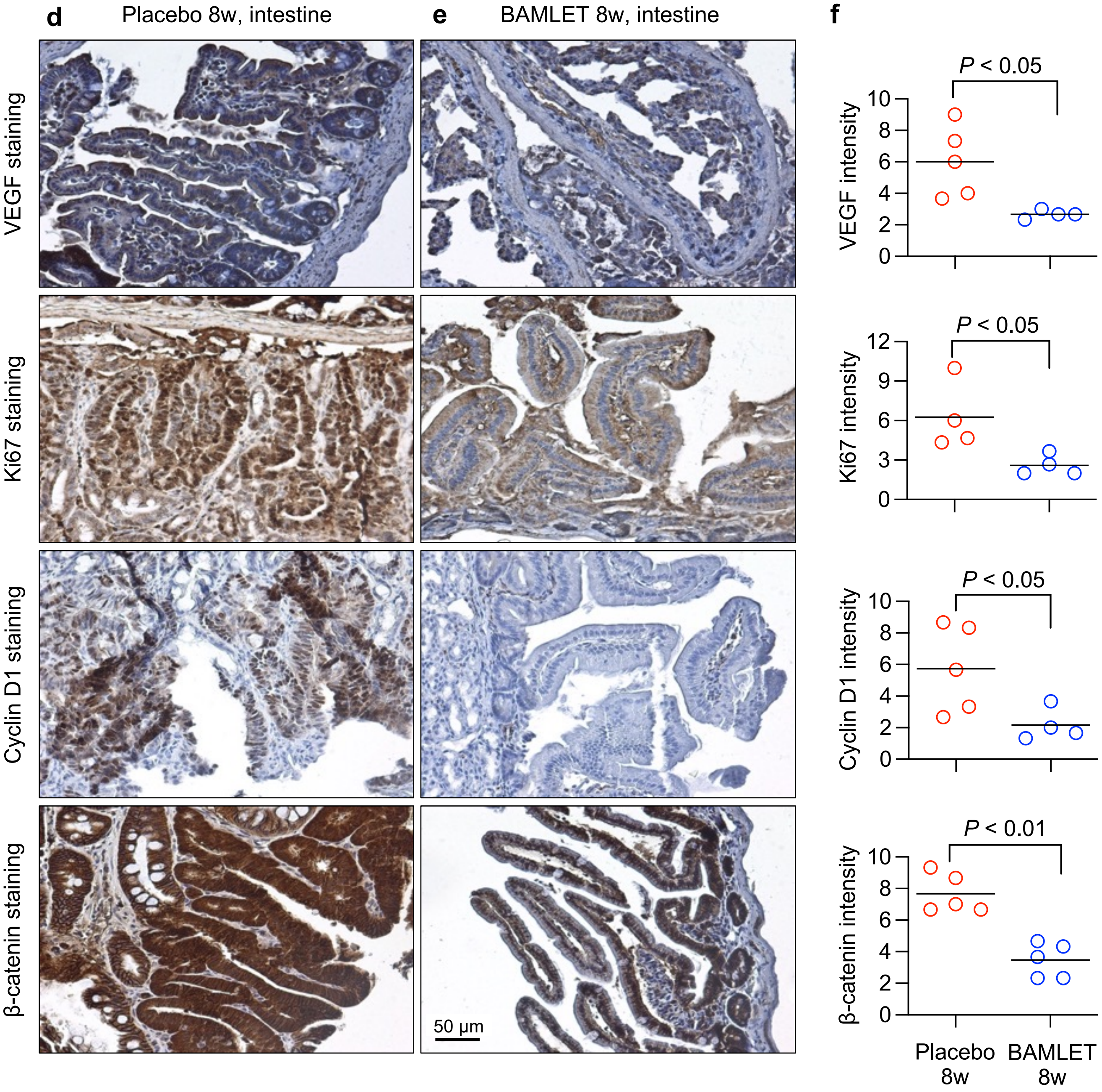

d
Placebo 8w, intestine
e
BAMLET 8w, intestine
f
VEGF staining
Ki67 staining
Cyclin D1 staining
β-catenin staining
50 μm
P < 0.05
VEGF intensity
10
8
6
4
2
0
P < 0.05
Ki67 intensity
12
9
6
3
0
P < 0.05
Cyclin D1 intensity
10
8
6
4
2
0
P < 0.01
β-catenin intensity
10
8
6
4
2
0
Placebo 8w
BAMLET 8w

Fig. S5

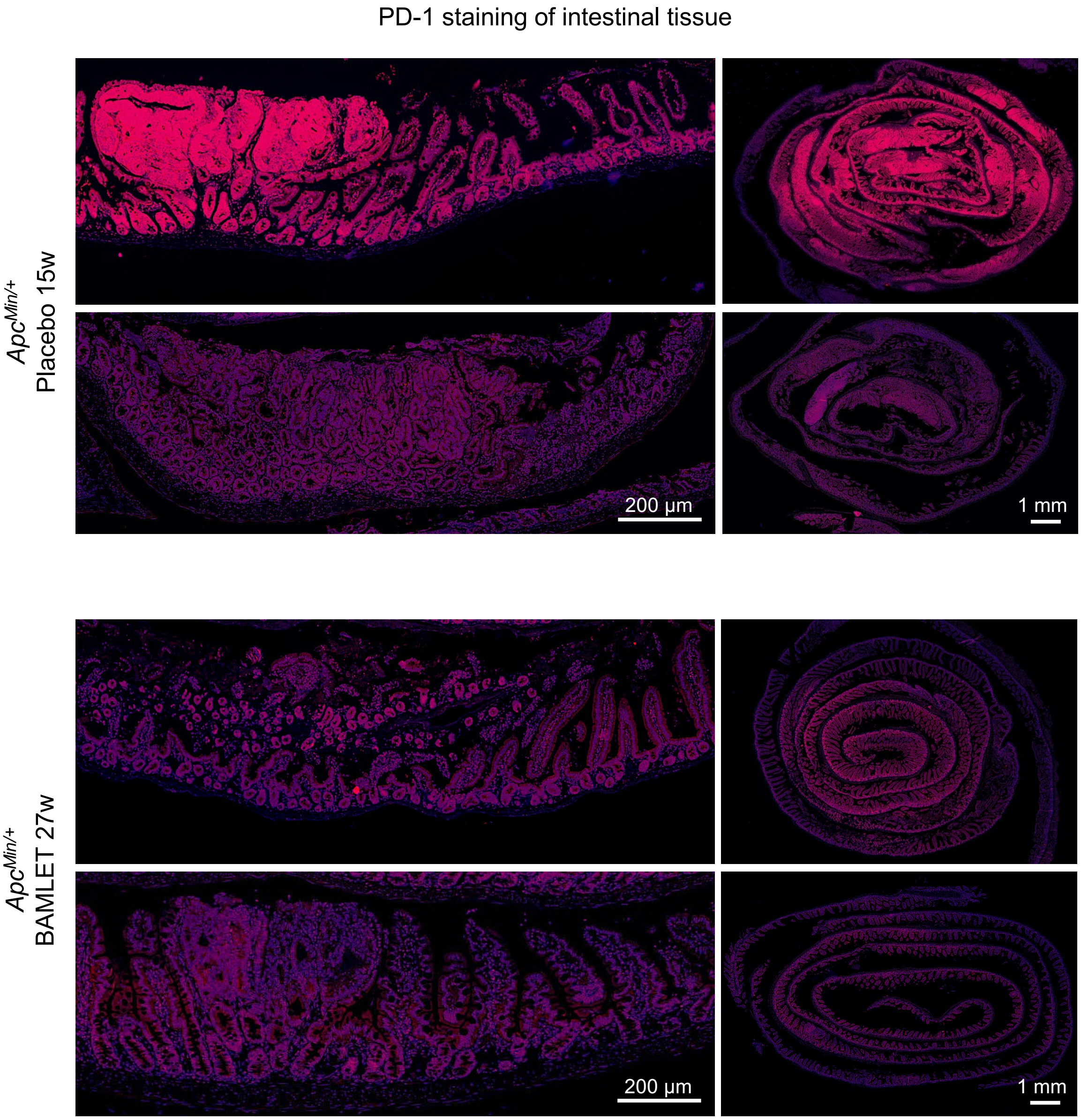
PD-1 staining of intestinal tissue
ApcMin/+
Placebo 15w
200 µm
1 mm
ApcMin/+
BAMLET 27w
200 µm
1 mm

Fig. S6

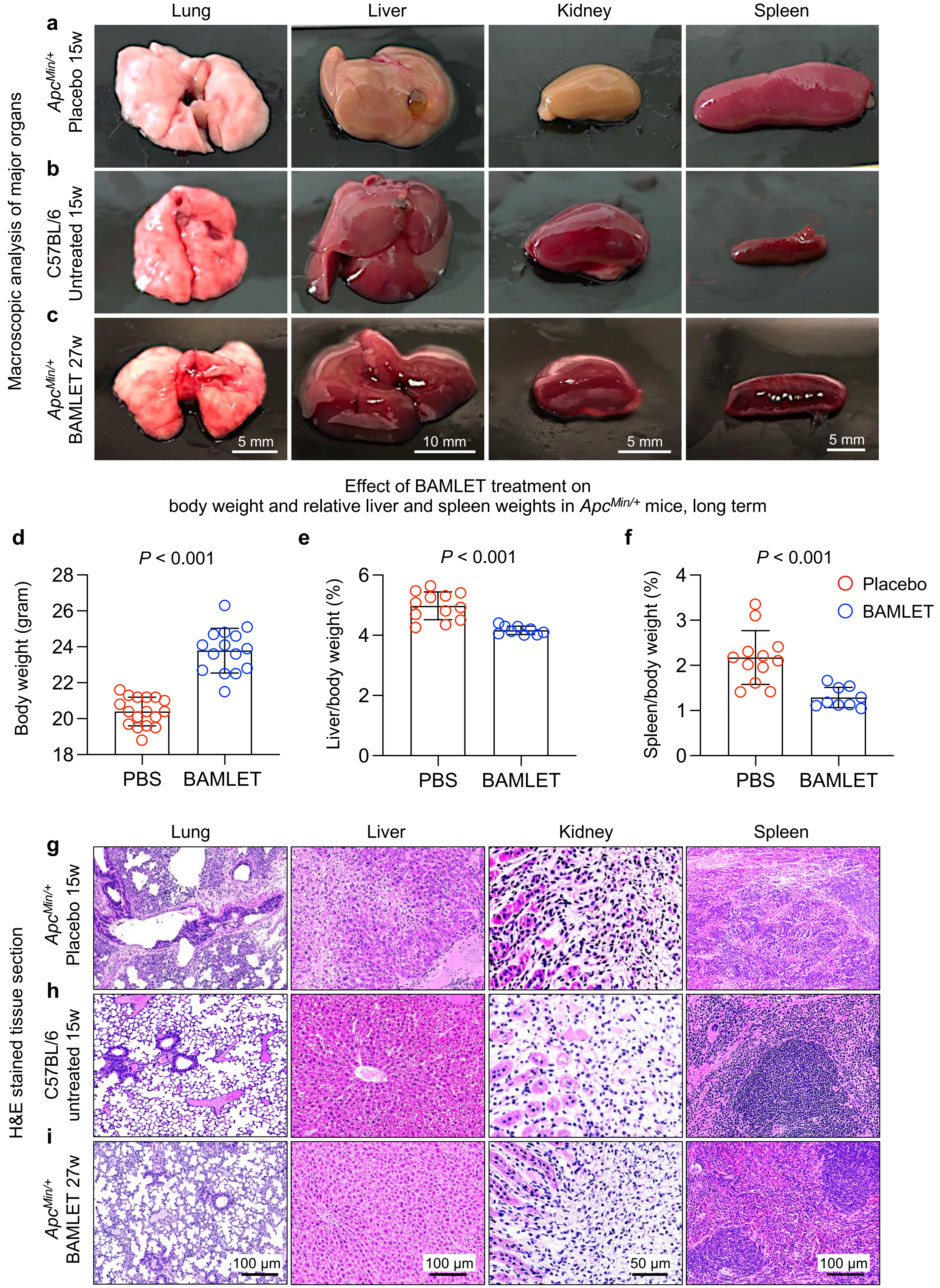

Macroscopic analysis of major organs
Lung
Liver
Kidney
Spleen
a
ApcMin/+ Placebo 15w
b
C57BL/6 Untreated 15w
c
ApcMin/+ BAMLET 27w
5 mm
10 mm
5 mm
5 mm
Effect of BAMLET treatment on
body weight and relative liver and spleen weights in ApcMin/+ mice, long term
d
P < 0.001
Body weight (gram)
28
26
24
22
20
18
PBS
BAMLET
e
P < 0.001
Liver/body weight (%)
6
4
2
0
PBS
BAMLET
f
P < 0.001
Spleen/body weight (%)
4
3
2
1
0
PBS
BAMLET
Placebo
BAMLET
H&E stained tissue section
Lung
Liver
Kidney
Spleen
g
ApcMin/+ Placebo 15w
h
C57BL/6 untreated 15w
i
ApcMin/+ BAMLET 27w
100 µm
100 µm
50 µm
100 µm

Fig. S7

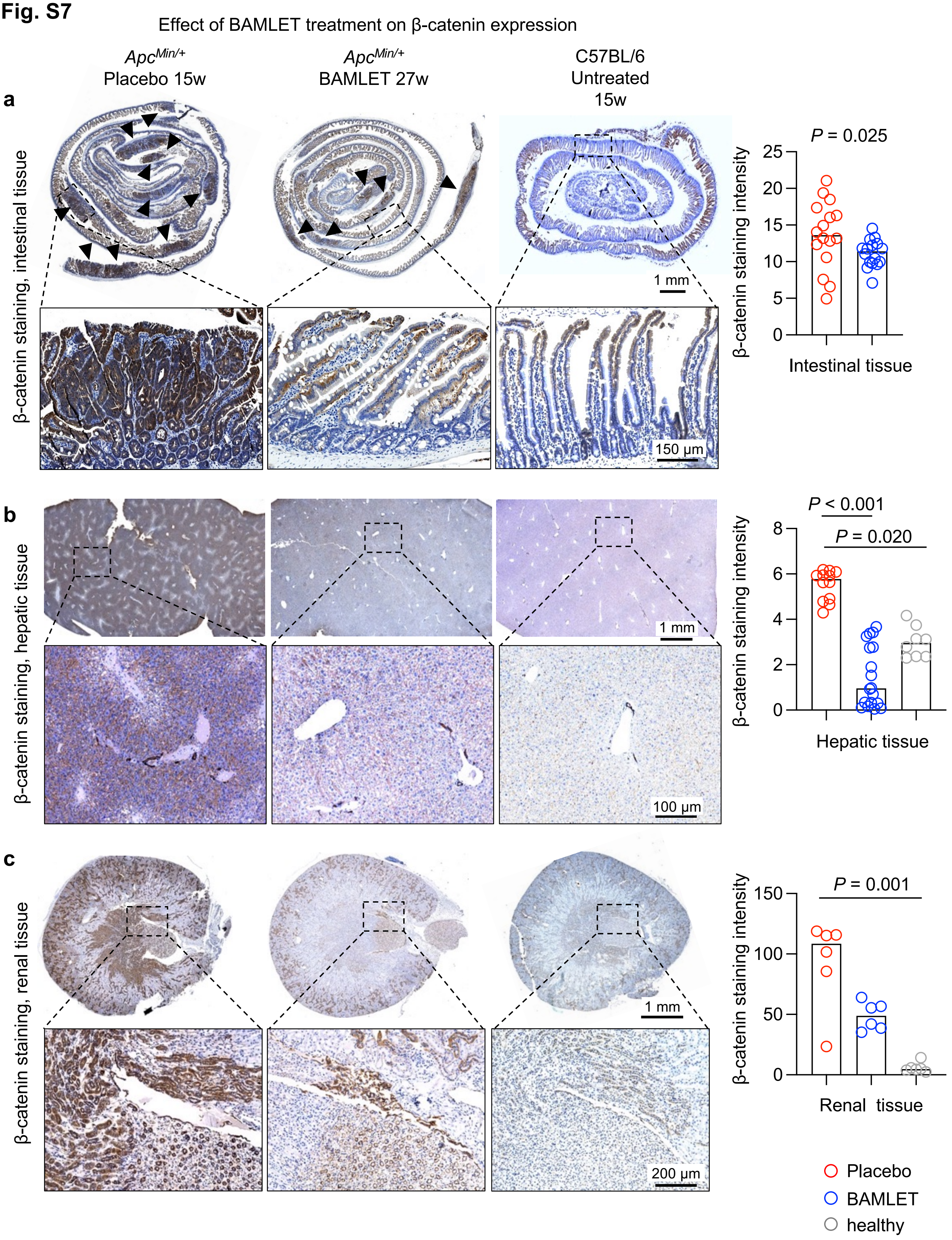

Effect of BAMLET treatment on β-catenin expression
ApcMin/+
Placebo 15w
ApcMin/+
BAMLET 27w
C57BL/6
Untreated
15w
a
β-catenin staining, intestinal tissue
1 mm
150 μm
P = 0.025
β-catenin staining intensity
25
20
15
10
5
0
Intestinal tissue
b
β-catenin staining, hepatic tissue
1 mm
100 μm
P < 0.001
P = 0.020
β-catenin staining intensity
8
6
4
2
0
Hepatic tissue
c
β-catenin staining, renal tissue
1 mm
200 μm
P = 0.001
β-catenin staining intensity
150
100
50
0
Renal tissue
Placebo
BAMLET
healthy

Fig. S8

a

Gene expression comparing the BAMLET 27w vs. Placebo 15w in *Apc*$^{Min/+}$ mice

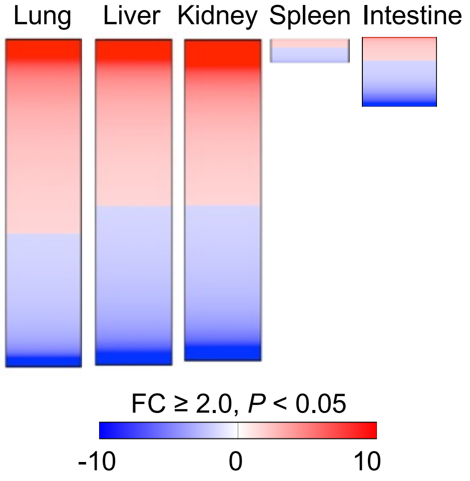

b

Total number of regulated genes by BAMLET 27w, *Apc*$^{Min/+}$ mice

	Liver	Kidney	Lung	Spleen	Intestine
Up	2421	2407	2724	91	112
Down	2291	2238	1851	159	216

Regulation of Wnt/β-catenin signalling pathway, BAMLET 27w vs. Placebo 15w, *Apc*$^{Min/+}$ mice

c

Lung tissue

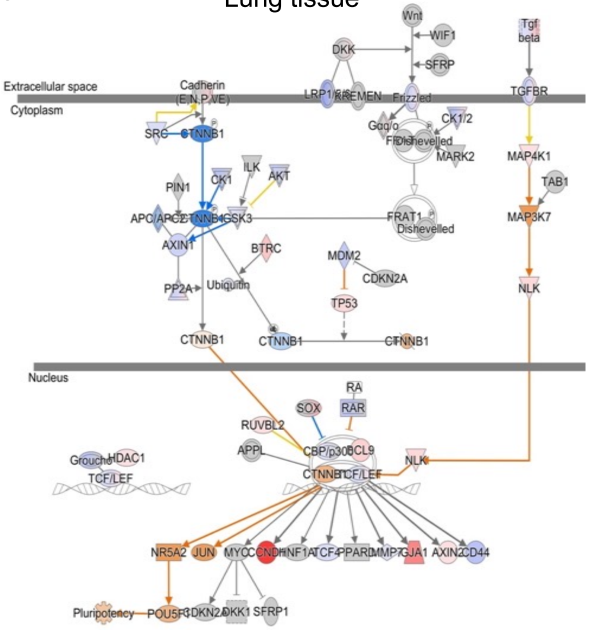

d

Liver tissue

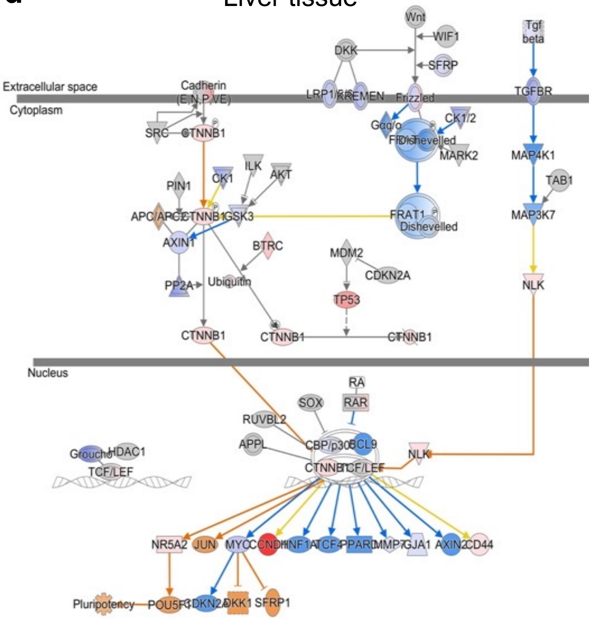

e

Kidney tissue

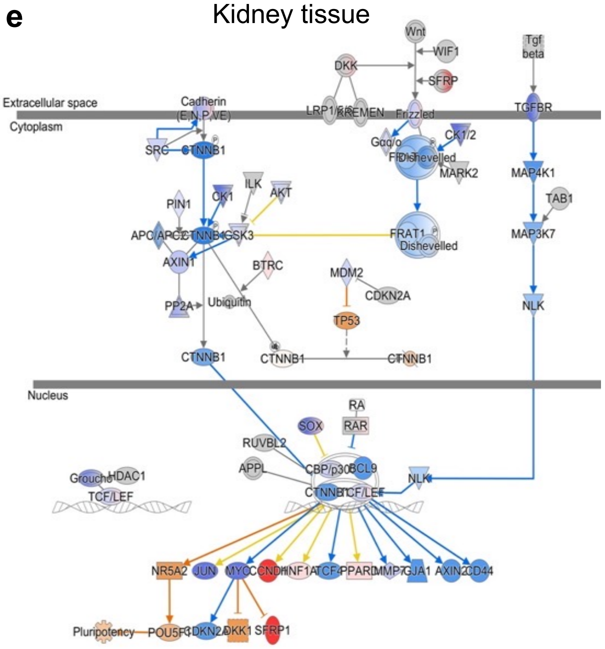

Fig. S9

BAMLET uptake by tumor tissues

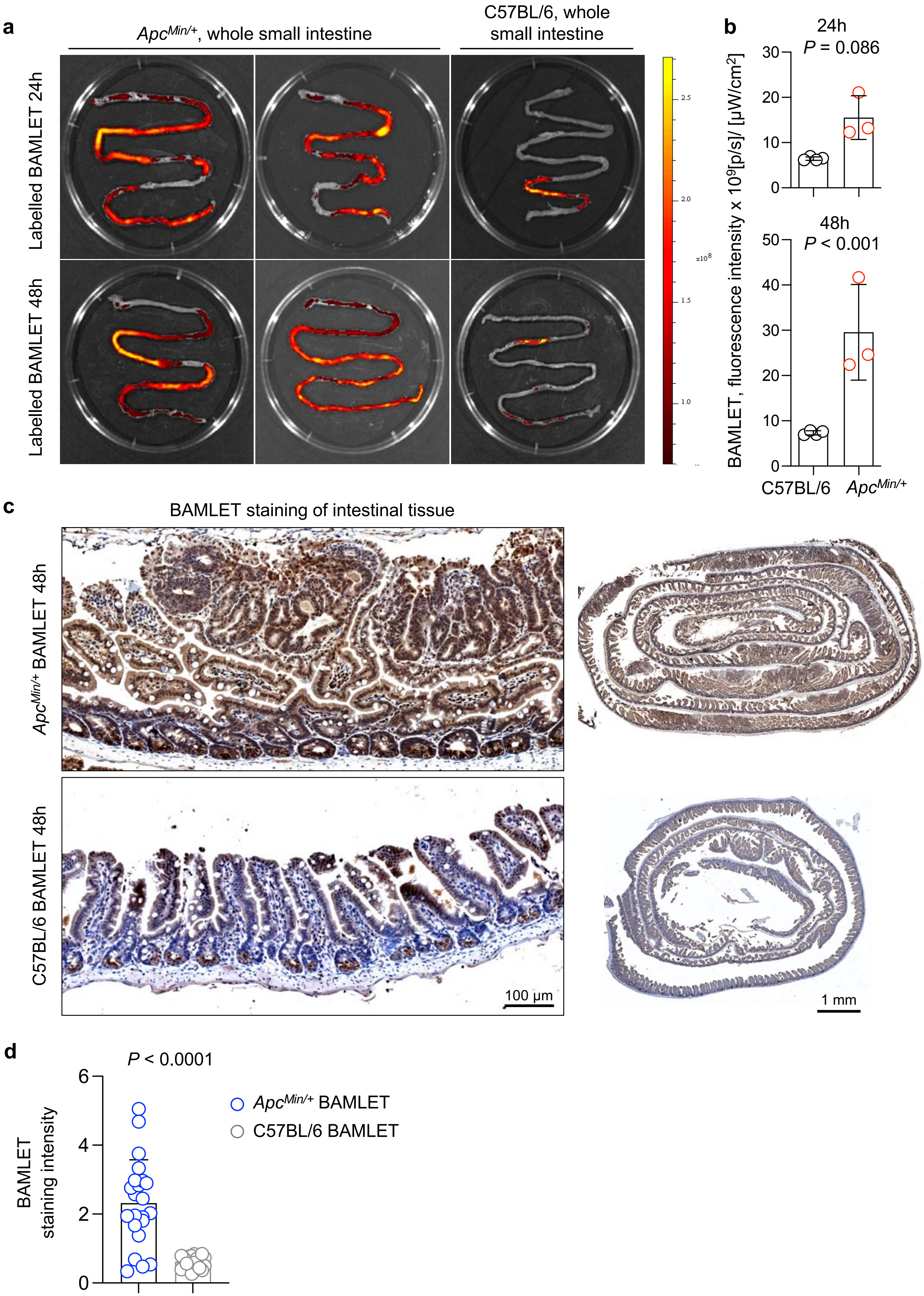

Fig. S10

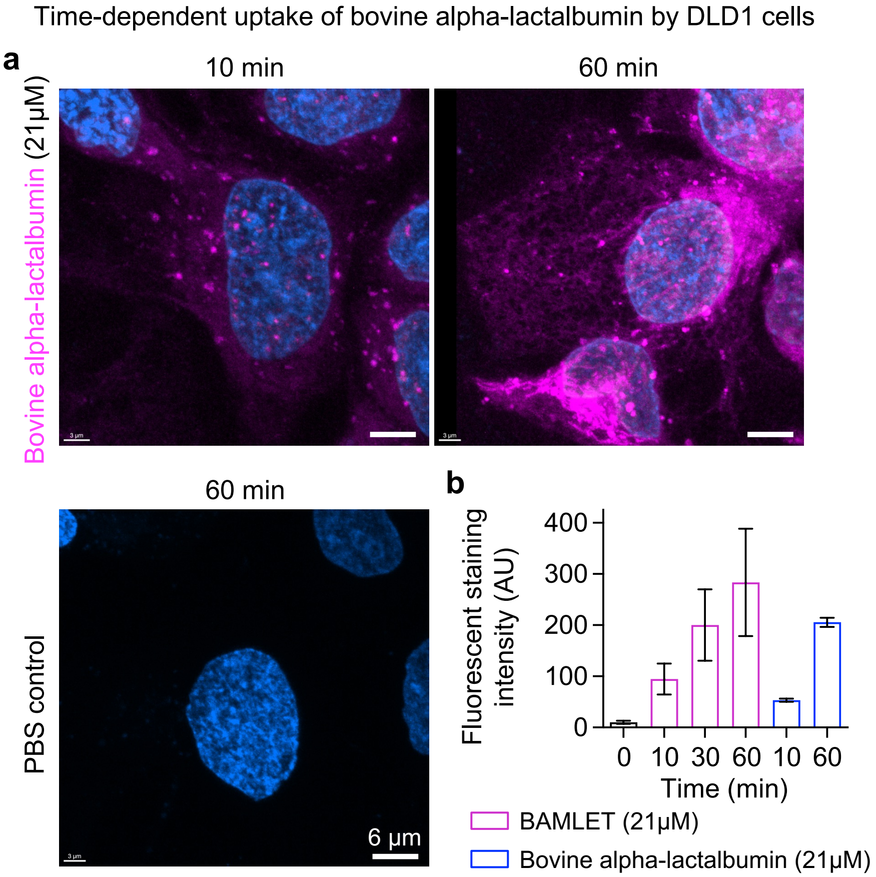

Time-dependent uptake of bovine alpha-lactalbumin by DLD1 cells
a
10 min
60 min
Bovine alpha-lactalbumin (21μM)
60 min
PBS control
6 μm
b
Fluorescent staining intensity (AU)
400
300
200
100
0
0
10
30
60
10
60
Time (min)
BAMLET (21μM)
Bovine alpha-lactalbumin (21μM)

Fig. S11

a

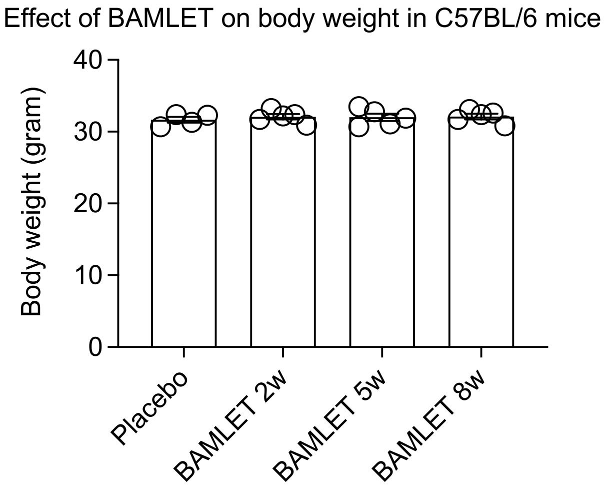
Effect of BAMLET on body weight in C57BL/6 mice
Body weight (gram)
40
30
20
10
0
Placebo
BAMLET 2w
BAMLET 5w
BAMLET 8w

b

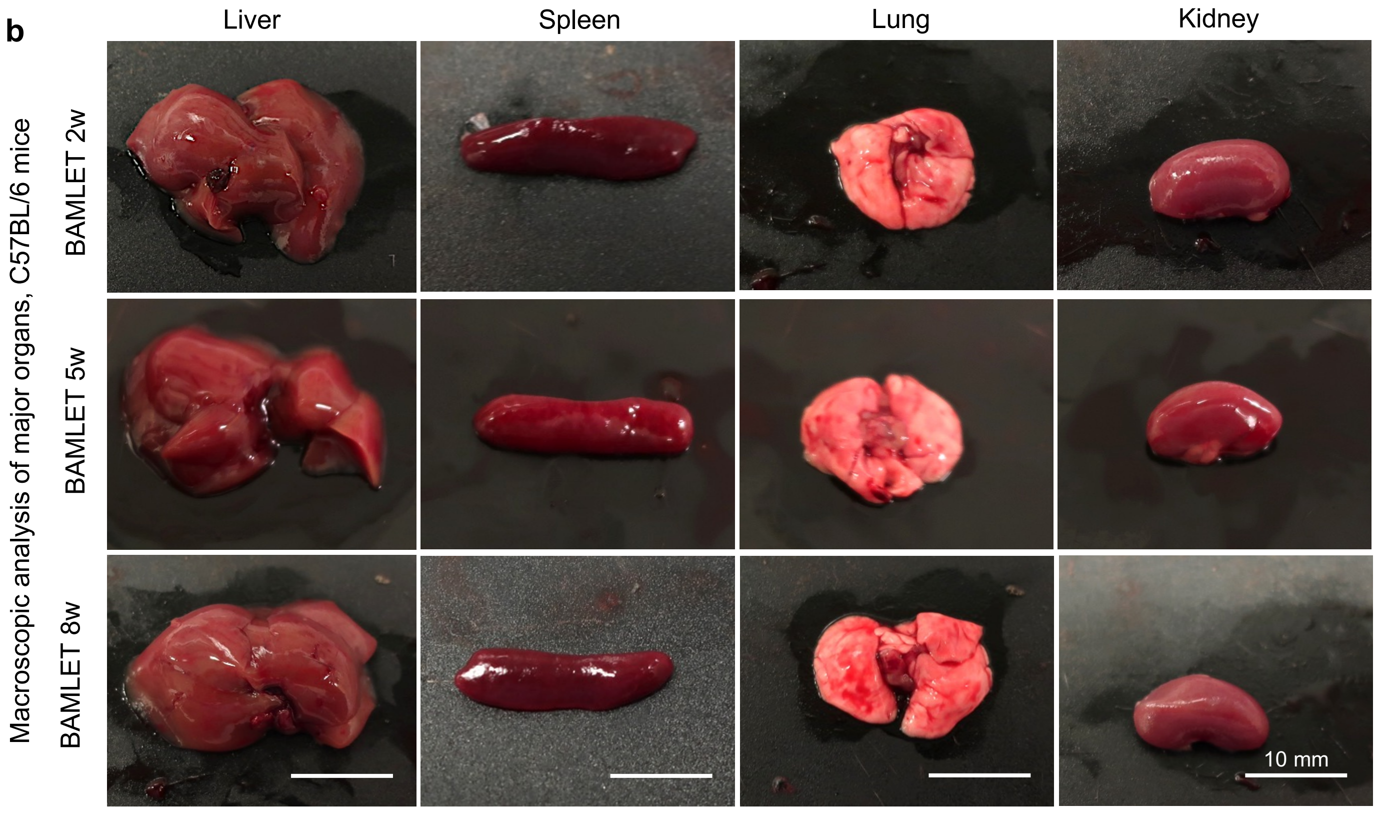
Liver
Spleen
Lung
Kidney
Macroscopic analysis of major organs, C57BL/6 mice
BAMLET 2w
BAMLET 5w
BAMLET 8w
10 mm

Fig. S12

Regulation of Glucose Metabolism pathway

a

C57BL/6 mice, BAMLET vs. Placebo, 5w

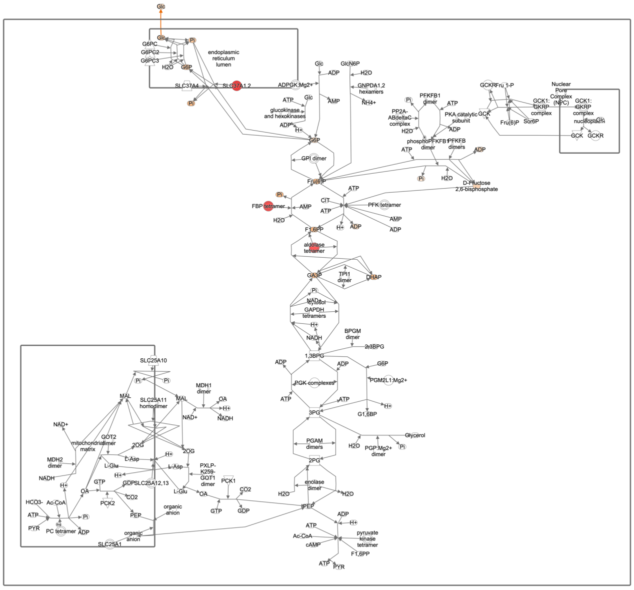

Apc$^{Min/+}$ mice, BAMLET vs. Placebo, 5w

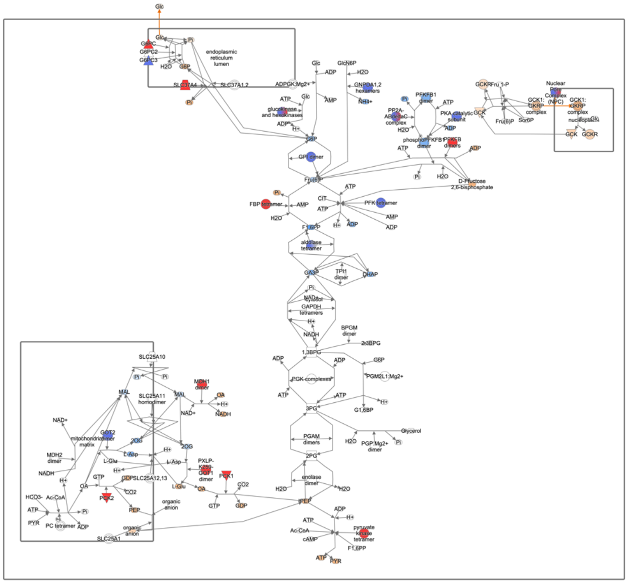

b Regulation of Glycolysis pathway

C57BL/6 mice, BAMLET vs. Placebo, 5w

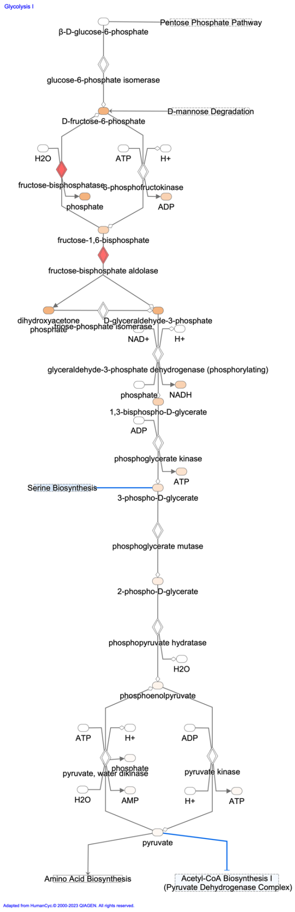

Apc$^{Min/+}$ mice, BAMLET vs. Placebo, 5w

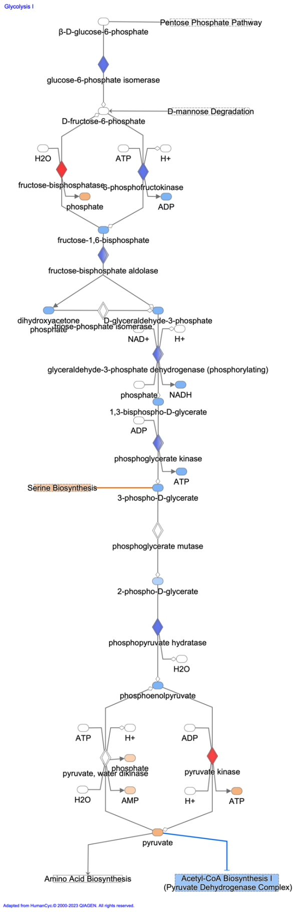

c Regulation of Gluconeogenesis pathway

C57BL/6 mice, BAMLET vs. Placebo, 5w

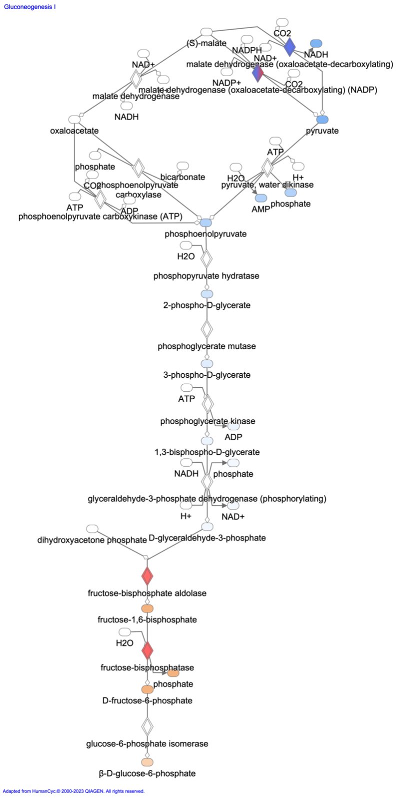

Apc$^{Min/+}$ mice, 5w BAMLET vs. Placebo, 5w

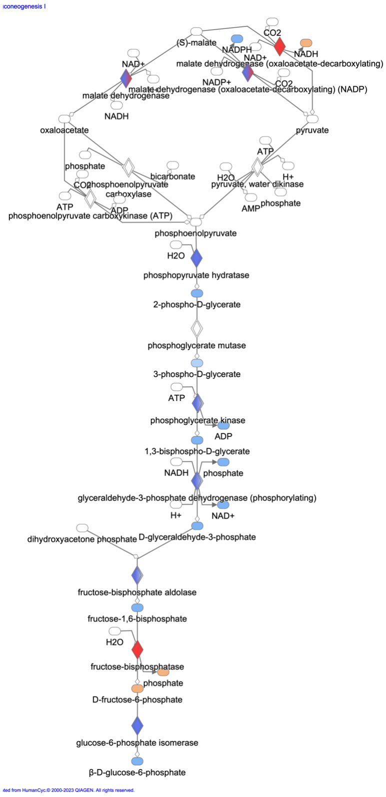

Fig. S12

d

Regulation of HIF1α signaling pathway,
C57BL/6 mice, BAMLET vs. Placebo, 5w

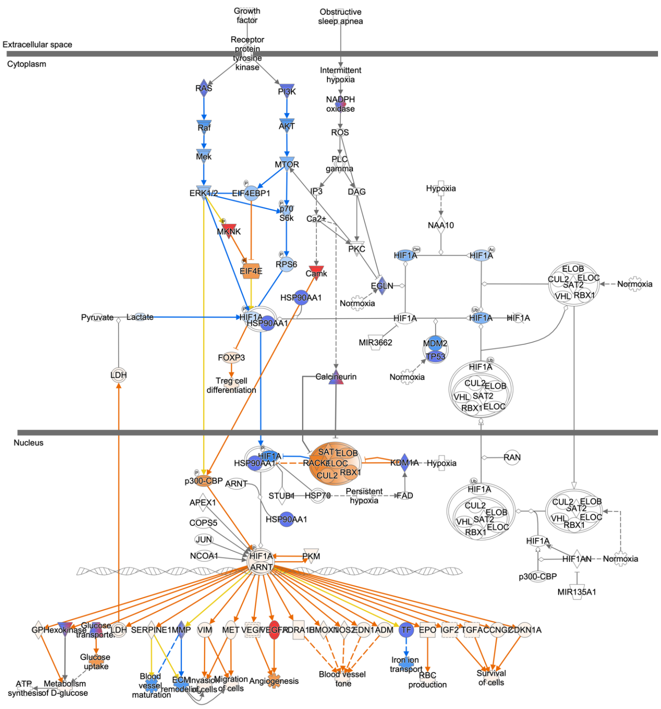

Regulation of HIF1α Signaling pathway,
Apc$^{Min/+}$ mice, BAMLET vs. Placebo, 5w,

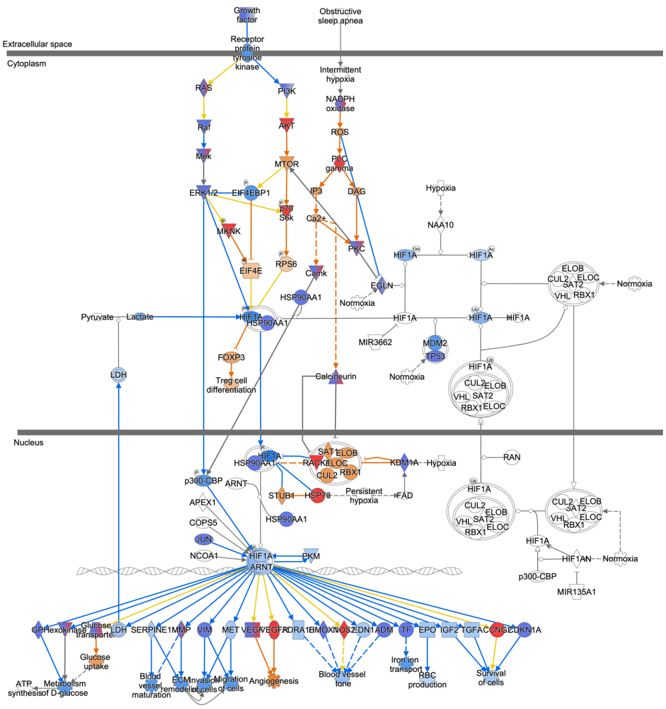

e

Regulation of PPAR signaling pathway,
C57BL/6 mice, BAMLET vs. Placebo, 5w

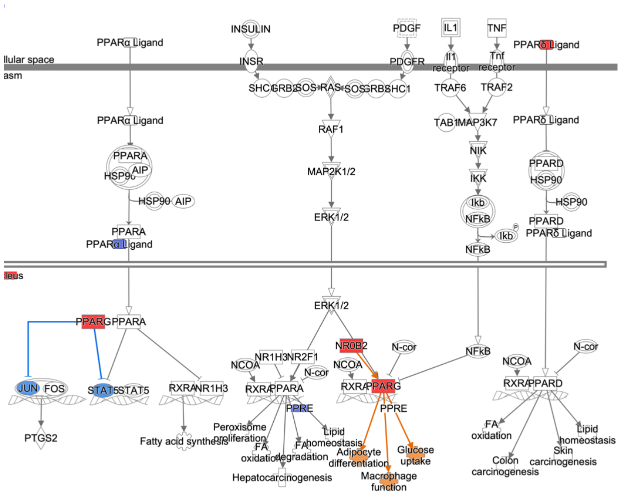

Regulation of PPAR Signaling pathway,
Apc$^{Min/+}$ mice, BAMLET vs. Placebo, 5w,

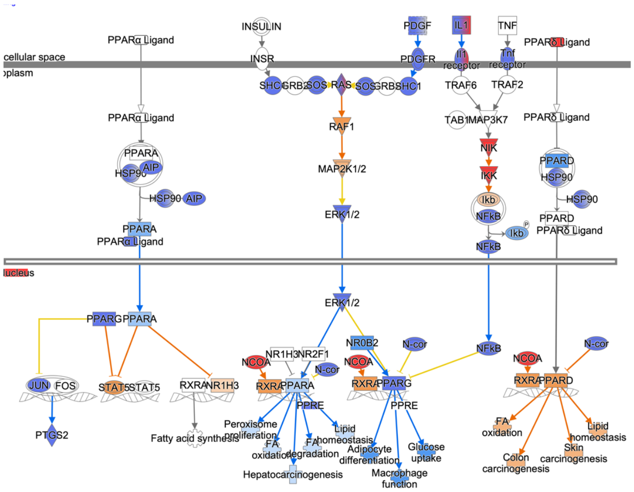

Supplement: Supplementary file 1 — Supplementary Information 1. [file 41598_2024_54040_MOESM1_ESM.pdf]
