# Supplementary material for: BAMLET administration via drinking water inhibits intestinal tumor development and promotes long-term health
Source: Sci Rep. 2024 Feb 15;14:3838. doi: 10.1038/s41598-024-54040-w (PMC10869698; doi:10.1038/s41598-024-54040-w)
Supplement: Supplementary file 2 — Supplementary Information 2. [file 41598_2024_54040_MOESM2_ESM.docx]

**Supplementary Fig. S1.** **Supplementary data for Main Fig. 1. BAMLET treatment delayed tumor progression in *Apc^Min/+^* mice.**

**a-e**, Data from *Apc^Min/+^* mice treated by oral gavage and followed for two weeks post-treatment (2w) (two experiments; PBS/placebo: *n* = 9, BAMLET: *n* = 10). **f-j**, Data from *Apc^Min/+^* mice treated by oral gavage and followed for five weeks post treatment (5w) (two experiments; PBS/placebo: *n* = 10, BAMLET: *n* = 10). **k-o**, Data from *Apc^Min/+^* mice that received BAMLET or PBS in the drinking water for eight weeks (8w) (two experiments; PBS/placebo: *n* = 13, BAMLET: *n* = 15). **a**, **f**, **k**, Dissection photomicrographs of small intestinal segments showing tumors (arrowheads) in the placebo-treated or the BAMLET-treated *Apc^Min/+^* mice. Representative image, *n* = two experiments, 4 mice per group. **b**, **g**, **l**, Methylene blue-stained whole mounts of the dissected intestinal segments (arrowheads, tumors). Representative image, *n* = two experiments, 4 mice per group. **c**, **h**, **m**, Polyp number in the BAMLET-treated compared to the placebo-treated *Apc^Min/+^* mice at 2w, 5w and 8w. **d**, **i**, **n**, Size distribution of polyps in the BAMLET-treated compared to the placebo-treated *Apc^Min/+^* mice at 2w, 5w and 8w (< 0.5 mm, 0.5-2 mm, > 2 mm). **e**, **j**, **o**, H&E-stained intestinal Swiss roll sections in the BAMLET-treated compared to the placebo-treated *Apc^Min/+^* mice, two experiments, *n* = 4 mice per group. Data are presented as the means ± S.E.M.s from two experiments and analyzed by two-way ANOVA with Šídák's multiple comparisons test (**d**, **i**, **n**) or Student’s *t* test (**c**, **h**, **m**). Scale bars = 3 mm.

**Supplementary Fig. S2.** **Supplementary data for Main Fig. 2. Inhibition of colon cancer-related genes in the BAMLET-treated mice.**

**a**, Heatmaps of colon cancer-related genes in *Apc^Min/+^* mice that received BAMLET by gavage (two or five weeks post-treatment, 2w or 5w) or in the drinking water (eight weeks, 8w) compared to the placebo-treated *Apc^Min/+^* mice. (red: upregulated genes, blue: downregulated genes, black: unregulated genes, cutoff FC ≥ 2.0 compared to the healthy intestinal tissue, *n* = 1 RNA sample per group). **b**, Number of colon cancer-related genes. **c**, Top regulated colon cancer-related genes identified by biofunction analysis.

**Supplementary Fig. S3. Supplementary data for Main Fig. 3. Reprogramming of cancer gene expression in the BAMLET-treated intestinal tissue.**

**a**, Heatmap comparing intestinal the gene expression profiles of the BAMLET-treated (10 mg/200 μl of BAMLET twice daily by oral gavage) and the placebo-treated (200 μl of PBS twice daily by oral gavage) *Apc^Min/+^* mice (red: upregulated genes, blue: downregulated genes, black: nonregulated genes, cutoff FC ≥ 2.0 BAMLET compared to the placebo group, *n* = 1 RNA sample per group). **b**, Venn diagram identifying genes regulated at all time points, in the three treatment groups compared to the placebo-treated *Apc^Min/+^* mice. **c**, Network analysis of shared genes revealed major treatment effects. Wnt/β-catenin signaling was inhibited in all the BAMLET-treated *Apc^Min/+^* mice. **d**, Genes in the tumor microenvironment network were broadly inhibited, potentially reducing proliferation, angiogenesis, metastasis, and the PD-1 pathway. Blue and orange represent inhibition and activation, whereas red and blue represent upregulation and downregulation, respectively. The intestinal RNA in the 5w group was from an outlier with the lowest treatment effect (experiment 1, highest number of polyps, 5w, Supplementary Fig. S1).

**Supplementary Fig. S4. Supplementary data for Main Fig. 4. Tumor markers in BAMLET-treated *Apc^Min/+^* mice compared to the placebo group.**

**a**, **b** Immunohistochemistry of intestinal sections stained for the tumor markers VEGF, Ki67, cyclin D1 and β-catenin. The BAMLET-treated (**b**) compared to the placebo-treated (**a**) *Apc^Min/+^* mice, five weeks (5w) after oral gavage. Representative sections, *n* = 5 mice per group. **c**, Quantification of the staining in (**a, b**). **d**, **e** Immunohistochemistry of intestinal sections stained for the tumor markers VEGF, Ki67, cyclin D1 and β-catenin. The BAMLET-treated (**e**) compared to the placebo-treated (**d**) *Apc^Min/+^* mice, eight weeks (8w) of BAMLET-supplemented drinking water. Representative sections, *n* = 5 mice per group. **f,** Quantification of the staining in (**d, e**). Data are presented as the means and analyzed by Mann-Whitney *U*-test (**c, f**), *n* = 5 mice per group. Scale bars = 50 μm.

**Supplementary Fig. S5. Supplementary data for Main Fig. 5. PD-1 staining of intestinal tissue sections.**

PD-1 staining of intestinal sections from the placebo-treated or BAMLET-treated *Apc^Min/+^* mice was performed with anti-PD-1 antibodies (magenta = PD-1, blue = DAPI). Representative sections, *n* = 3 mice per group. Scale bars = 200 μm (close-up), 1 mm (whole tissue).

**Supplementary Fig. S6. Supplementary data for Main Fig. 4. Evidence of extra-intestinal disease** **in *Apc^Min/+^* mice receiving placebo and protection in BAMLET treated mice.**

**a-c** Macroscopic appearance of the lungs, livers, kidneys and spleens obtained from the placebo-treated *Apc^Min/+^* mice (**a**) compared to the healthy C57BL/6 mice at sacrifice after long-term follow-up (15 weeks, 15w) (**b**). Changes in tissue morphology indicated systemic involvement in the placebo group. **c,** These effects were reduced in the group that received BAMLET in drinking water (27 weeks, 27w). **d-f**, Body weights, relative liver and spleen weights (net organ weight / body weight) of the BAMLET-treated *Apc^Min/+^* mice compared to the placebo-treated mice. Significant increase in body weight (**d**) and significant decrease in relative liver (**e**) and spleen (**f**) weights were observed for the BAMLET-treated mice compared to the placebo-treated mice. Data are presented as the means ± S.D. and analyzed by unpaired t-test (**d-f**), *n* = 9 - 12 mice per group. **g-i,** Tissue analysis of H&E-stained lung, liver, kidney and spleen sections. Representative sections, *n* = 4 mice per group. Scale bar = 50 μm (kidney), 100 μm (lung, liver and spleen).

**Supplementary Fig. S7. Supplementary data for Main Fig. 4. Reduction in β-catenin staining in intestinal, hepatic and renal tissues of BAMLET-treated mice.**

**a-c,** β-catenin staining was quantified in tissue sections from the intestine, liver and kidney of the placebo-treated and the BAMLET-treated *Apc^Min/+^* mice and compared to that of healthy C57BL/6 controls. Representative sections, *n* = 4 mice per group, 4 areas per mouse (intestine); *n* = 2-3 mice per group, 6 areas per mouse (liver); *n* = 2 mice per group, 3 areas per mouse (kidney). Reduced β-catenin staining in intestinal (**a**), hepatic (**b**) and renal (**c**) tissues from the *Apc^Min/+^* mice that received BAMLET-supplemented drinking water long-term is consistent with the inhibition of Wnt/β-catenin signaling outside of the intestinal compartment. Data are presented as the medians and analyzed by one-way ANOVA with Šídák’s multiple comparisons test (**a**) and two-tailed Kruskal–Wallis test with Dunn’s correction (**b**, **c**). Scale bars = 1 mm (whole tissue), 150 μm (close-up) (intestine) (**a**); 1 mm (whole tissue), 100 μm (close-up) (liver) (**b**); 1 mm (whole tissue), 200 μm (close-up) (kidney) (**c**).

**Supplementary Fig. S8. Supplementary data for Main Fig. 4.** **Gene expression analysis of lungs, livers and kidneys from** **BAMLET-treated compared to placebo-treated *Apc^Min/+^*** **mice.**

**a**, Gene expression analysis of lungs, livers, kidneys and spleens from the BAMLET-treated and the placebo-treated *Apc^Min/+^* mice. Heatmap comparing gene expression profiles (red: upregulated genes, blue: downregulated genes, cutoff FC ≥ 2.0, *P* < 0.05, compared to placebo, *n* = 2 RNA samples per group). **b,** Total number of regulated genes in lung, liver, kidney and spleen tissues of the BAMLET-treated mice compared to the placebo-treated mice (cutoff FC ≥ 2.0 compared to the placebo-treated group) **c-e,** The Wnt/β-catenin signaling pathway was strongly decreased in lungs (**c**), livers (**d**) and kidneys (**e**) from the BAMLET-treated mice compared to the placebo-treated *Apc^Min/+^* mice. Blue and orange represent inhibition and activation, whereas red and blue represent upregulation and downregulation, respectively.

**Supplementary Fig. S9. Retention of BAMLET in tumor tissue, as shown by *in vivo* imaging of BAMLET and immunohistochemistry of intestinal tissue sections.**

**a**, *In vivo* imaging of intestines from the tumor-bearing *Apc^Min/+^* mice or C57BL/6 mice (both 18 weeks old) exposed by gavage to VivoTag 680-labeled BAMLET. Retention of BAMLET in the tumor-bearing *Apc^Min/+^* mice after 24 hours (*n* = 3) and 48 hours (*n* = 3) compared to that in healthy C57BL/6 mice (two experiments; *n* = 8). **b**, Quantification of the fluorescence intensity in intestinal sections at 24 hours (upper) and 48 hours (lower) after BAMLET administration. **c**, BAMLET staining in the tumor area of intestinal sections from (**a**) using immunohistochemistry. Representative sections, *n* = 3 mice per group. **d**, Quantification of the BAMLET staining from (**c**). Data are presented as the means ± S.E.M.s and analyzed by Student’s *t* test. Scale bars = 100 μm (close-up), 1 mm (whole tissue mount) (**c**).

**Supplementary Fig. S10. Supplementary data for Main Fig. 7. Analysis of cells treated with bovine alpha-lactalbumin compared to BAMLET.**

**a**, Live-cell confocal images showing the time-dependent uptake of Janelia Fluor-549 labeled bovine alpha-lactalbumin (21 μM, magenta) by DLD1 human colorectal adenocarcinoma cells. Nuclei were counterstained with Hoechst (blue). **b**, Quantification of the bovine alpha-lactalbumin cellular uptake in (**a**) compared to BAMLET (*n* = 50 cells per group). Significant uptake of bovine alpha-lactalbumin occurred more slowly and to a lower extent than for the complex. Data are presented as the means ± S.E.M.s, analyzed by two-way ANOVA with Šídák's multiple comparisons test (**b**). Scale bar = 6 μm (**a**).

**Supplementary Fig. S11. Supplementary data for Main Fig. 8. Lack of toxicity in healthy C57BL/6 mice exposed to BAMLET.**

Following the protocols in Fig. 1, BAMLET was administered to healthy C57BL/6 mice by gavage and followed two weeks (2w), or five weeks (5w) post treatment (*n* = 5 mice per group) or was administered BAMLET in the drinking water for eight weeks (8w; 20 mg of in 5 ml PBS daily, *n* = 5). **a,** Body weights of the BAMLET-treated C57BL/6 mice compared to the placebo-treated mice. No significant changes were observed for the BAMLET-treated mice compared to the placebo-treated mice. **b,** Intact macroscopic appearance of the livers, spleens, lungs, and kidneys obtained from the BAMLET-treated C57BL/6 mice at sacrifice. Representative images, *n* = 5 mice per group. Data are presented as the means ± S.E.M.s, analyzed by one-way ANOVA with Šídák's multiple comparisons test (**a**). Scale bar = 10 mm (**b**).

**Supplementary Fig. S12. Supplementary data for Main Fig. 8. Effects of BAMLET on genes regulating metabolic functions in healthy C57BL/6 mice.**

**a**, Effects of BAMLET treatment on the Glucose Metabolism pathway in C57BL/6 mice and *Apc^Min^*^/+^ mice (five weeks (5w) post treatment) (*n* = 2 RNA samples per group). **b**, Effects of BAMLET treatment on the Glycolysis pathway in C57BL/6 mice and *Apc^Min^*^/+^ mice (5w post treatment). **c**, Effects of BAMLET treatment on the Gluconeogenesis pathway in C57BL/6 mice and *Apc^Min^*^/+^ mice (5w post treatment). **d,** Effects of BAMLET treatment on the HIF1α signaling in C57BL/6 mice and *Apc^Min^*^/+^ mice (5w post treatment). **e**, Effects of BAMLET treatment on the lipid metabolism in C57BL/6 mice and *Apc^Min^*^/+^ mice (5w post treatment). Blue and orange represent inhibition and activation, whereas red and blue represent upregulation and downregulation, respectively.
